# Phenoxyacetohydrazide Schiff Bases: *β*-Glucuronidase Inhibitors

**DOI:** 10.3390/molecules19078788

**Published:** 2014-06-25

**Authors:** Waqas Jamil, Shagufta Perveen, Syed Adnan Ali Shah, Muhammad Taha, Nor Hadiani Ismail, Shahnaz Perveen, Nida Ambreen, Khalid M. Khan, Muhammad I. Choudhary

**Affiliations:** 1 H. E. J. Research Institute of Chemistry, International Center for Chemical and Biological Sciences, University of Karachi, Karachi 75270, Pakistan; E-Mails: waqas143kh@yahoo.com (W.J.); sarosh.iqbal@yahoo.com (S.P.); nida_mishi2002@yahoo.com (N.A.); hassaan2@super.net.pk (M.I.C.); 2 Institute of Advance Research Studies in Chemical Sciences, University of Sindh Jamshoro, Hyderabad 76080, Pakistan; 3 Faculty of Pharmacy, Universiti Teknologi MARA (UiTM), Puncak Alam Campus, Bandar Puncak Alam, Selangor Darul Ehsan 42300, Malaysia; E-Mail: benzene301@yahoo.com or syedadnan@salam.uitm.edu.my; 4 Atta-ur-Rahman Institute for Natural Products Discovery (AuRIns), Level 9, FF3, Universiti Teknologi MARA (UiTM), Puncak Alam Campus, Bandar Puncak Alam, Selangor Darul Ehsan 42300, Malaysia; E-Mails: taha_hej@yahoo.com (M.T.); norhadiani@puncakalam.uitm.edu.my (N.H.I.); 5 Faculty of Applied Science UiTM, Shah Alam, Selangor 40450, Malaysia; 6 PCSIR Laboratories Complex, Shahrah-e-Dr. Salimuzzaman, Karachi 75280, Pakistan; E-Mail: shamim_302@yahoo.com

**Keywords:** phenoxyacetohydrazide, Schiff bases, *β*-glucouoronidase, glucuronosyl-*O*-bonds, D-saccharic acid-1,4-lactone

## Abstract

Phenoxyacetohydrazide Schiff base analogs **1**–**28** have been synthesized and their *in vitro* *β*-glucouoronidase inhibition potential studied. Compounds **1** (IC_50_ = 9.20 ± 0.32 µM), **5** (IC_50_ = 9.47 ± 0.16 µM), **7** (IC_50_ = 14.7 ± 0.19 µM), **8** (IC_50_ = 15.4 ± 1.56 µM), **11** (IC_50_ = 19.6 ± 0.62 µM), **12** (IC_50_ = 30.7 ± 1.49 µM), **15** (IC_50_ = 12.0 ± 0.16 µM), **21** (IC_50_ = 13.7 ± 0.40 µM) and **22** (IC_50_ = 22.0 ± 0.14 µM) showed promising *β*-glucuronidase inhibition activity, better than the standard (D-saccharic acid-1,4-lactone, IC_50_ = 48.4 ± 1.25 µM).

## 1. Introduction

A range of bioactivities are reported for hydrazide-hydrazone compounds, such as antibacterial, anticonvulsant, antimalarial, analgesic, antiinflammatory, antiplatelets, antifungal, antituberculosis, and anticancer activities [1,2,3,4,5,6]. A variety of semicarbazones, thiosemicarbazones and guanyl hydrazones are found to be key compounds for drug design [7], for metal complexes [8], organocatalysis [9], and are used for the preparation of heterocyclic rings [10]. A few pyrazole carbohydrazide hydrazone derivatives [11] and novel 3-aryl-1-arylmethyl-1*H*-pyrazole-5-carbohydrazide hydrazones were found to be proliferation inhibitors of A549 cells [12,13] Some evidence proposes a pharmacophoric character for the hydrazone moiety present in phenylhydrazone derivatives in the inhibition of cyclooxygenase [14]. Antioxidant [15,16,17,18], antiglycation [19,20,21,22] and antileishmanial [23] activity have recently been reported, as well as applications in mass spectrometry [24].

The present work aimed to investigate the potential activity of a series of aryl hydrazide-hydrazones as *in vitro β*-glucouoronidase inhibitors. In our designed analogues substituted phenoxy-acetohydrazides were treated with different aromatic aldehydes to scrutinize their potential activity. The earlier reported literature [25] showed that *β*-glucouoronidase is a lysosomal enzyme, present in many organs like the spleen, kidney, lung, bile, serum and urine, *etc.*, where its specific task is to catalyze the cleavage of glucuronosyl-*O*-bonds [26,27,28]. It degrades glucuronic acid-containing glycosaminoglycans, like heparan sulfate, chondroitin sulfate and dermatan sulfate [29]. An elevated level of *β*-glucouoronidase was observed in various types of malignancies, such as breast, lung and gastrointestinal tract carcinomas, and melanomas. Its high expression also observed in bronchial tumors [30]. On the other hand, mucopolysaccharidosis type VII (MPS VII; Sly Syndrome) is caused by the deficiency of human *β*-glucuronidase [31]. The circulating level of *β*-glucuronidase is also useful as a lysosomal enzyme in children affected by leprosy. In borderline tuberculoid patients and lepromatous patients higher activity of this enzyme was also observed.

## 2. Results and Discussion

### 2.1. Chemistry

**Scheme 1:**
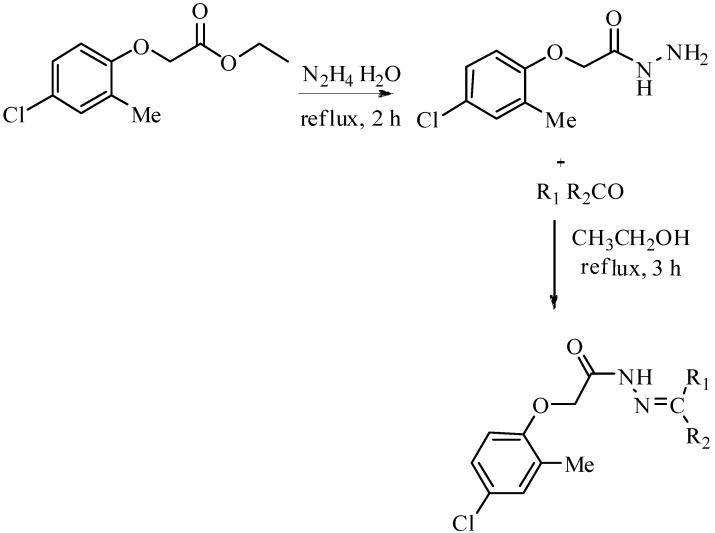
Synthetic scheme for benzohydrazide followed by synthesis of Schiff bases **1**–**28**.

Lead identification is a well defined tool in drug design and discovery. Our research group has been involved for a decade in lead discovery programs in search of novel therapeutic agents. We have earlier reported Schiff bases of different classes of organic compounds in the search for lead molecules with different biological activities [32,33,34]. Earlier, our group reported the leishmanicidal and *β*-glucurinodase inhibition potential of hydrazides derived from the corresponding esters [35,36,37,38]. In view of the formerly reported work we synthesized hydrazide Schiff bases and screened their potential biological activities [39,40,41,42]. Acylhydrazide Schiff base derivatives **1**–**28** were synthesized from an acylhyrazide by condensing it with different aromatic aldehydes and acetophenones under reflux conditions in ethanol for 2–3 h (Scheme 1). The crude products (Table 1) were further recrystallized from methanol and needle-like crystals were obtained in most of the cases. The starting acylhydrazide was synthesized from ester of ethyl 2-(4-chloro-2-methylphenoxy) acetate by refluxing with hydrazine hydrate.

**Table 1:** Synthesis of acylhydrazide Schiff base derivatives **1**–**28**.

Compound No.	R_1_	R_2_	Yield (%)	Compound No.	R_1_	R_2_		Yield (%)	
**1**	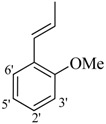	H	81	**15**	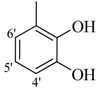	H		88	
**2**	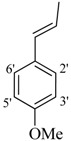	H	85	**16**	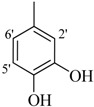	H		92	
**3**	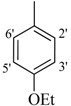	H	93	**17**	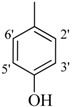	CH_3_		94	
**4**	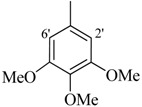	H	87	**18**	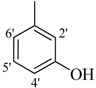	H		89	
**5**	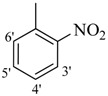	H	83	**19**	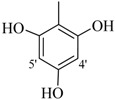	CH_3_		87	
**6**	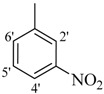	H	86	**20**	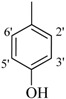	H		84	
**7**	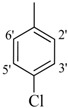	H	89	**21**	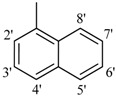	H		94	
**8**	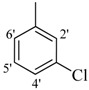	H	91	**22**	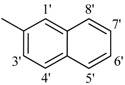	H		93	
**9**	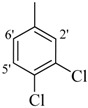	H	94	**23**	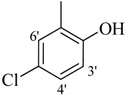	H		91	
**10**	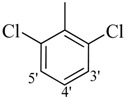	H	82	**24**	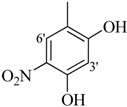	CH_3_		95	
**11**	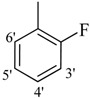	H	88	**25**	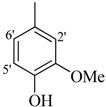	H		91	
**12**	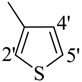	H	91	**26**	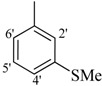	H		88	
**13**	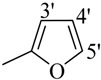	H	86	**27**	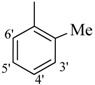	H		81	
**14**	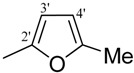	H	93	**28**	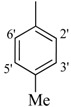	H		93	

### 2.2. β-Glucuronidase

Synthetic acyl hydrazides Schiff bases **1**–**28** were screened for their *in vitro* potential as *β*-glucoronidase inhibitors. The *in vitro β*-glucornidase inhibitory potential was evaluated by using the literature protocol [43]. Compounds **1**–**28** showed diversified *β*-glucoronidase inhibitory activities, with IC_50_ values ranging between 9.20–30.7 µM. Compounds **1**, **5**, **7**, **8**, **11**, **12**, **15**, **21**, and **22** showed excellent *β*-glucoronidase inhibitory activities, with IC_50_ values of 9.20 ± 0.32, 9.47 ± 0.16, 14.7 ± 0.19, 15.4 ± 1.56, 19. ± 0.62, 30.7 ± 1.49, 12.0 ± 0.16, 13.7 ± 0.40, and 22.0 ± 0.14 µM, respectively, and the remaining compounds exhibited no activity (Table 2).

**Table 2:** *In vitro β-*glucuronidase activity of compounds **1**–**28**.

Compounds	IC_50_ (μM ± SEM ^a^)	Compounds	IC_50_ (μM ± SEM ^a^)
**1**	9.20 ± 0.32	**15**	12.0 ± 0.16
**2**	NA ^b^	**16**	NA ^b^
**3**	NA ^b^	**17**	NA ^b^
**4**	NA ^b^	**18**	NA ^b^
**5**	9.47 ± 0.16	**19**	NA ^b^
**6**	NA ^b^	**20**	NA ^b^
**7**	14.7 ± 0.19	**21**	13.7 ± 0.40
**8**	15.4 ± 1.56	**22**	22.0 ± 0.14
**9**	NA ^b^	**23**	NA ^b^
**10**	NA ^b^	**24**	NA ^b^
**11**	19.6 ± 0.62	**25**	NA ^b^
**12**	30.7 ± 1.49	**26**	NA ^b^
**13**	NA ^b^	**27**	NA ^b^
**14**	NA ^b^	**28**	NA ^b^
D-saccharic acid-1,4-lactone ^c^	48.4 ± 1.25	**-**	-

SEM ^a^ is the standard error of the mean; NA ^b^ Not active; ^c^ standard inhibitor for *β*-glucuronidase.

**Figure 1:**
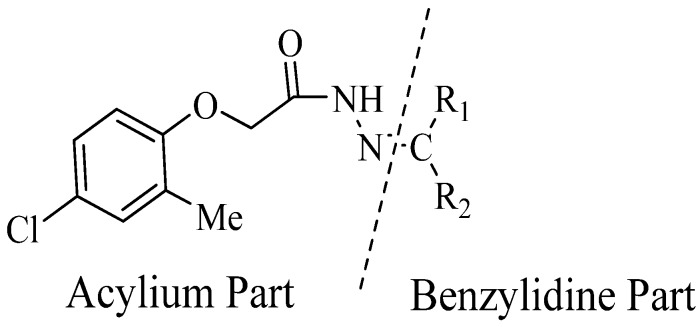
The two parts of molecule on which activity is based.

It was observed that both the substituents’ nature and their position at the benzilidine part have great importance in the *β*-glucoronidase inhibition activity of a compound, and apparently the acylium part does not take part in the activity (Figure 1).

The best activity was shown by compound **1** (IC_50_ = 9.20 ± 0.32 µM, fivefold better than the standard D-saccharic acid-1,4-lactone, IC_50_ value 48.4 ± 1.25 µM) which has a methoxy group at the *ortho* position. Surprisingly, a marked decline in activity (to the point of being inactive) was observed in analog **2** which has a methoxy residue at the *para* position instead of the *ortho* position as in compound **1**. This huge difference in the activities of compounds **1** and **2** clearly indicates that a specific group at a specific position of the benzylidine phenyl ring part plays a vital role in making a potent *β*-glucornidase inhibitor in this type of compounds. The excellent activity of compound **5** (IC_50_ = 9.47 ± 0.16 µM) having an *ortho* nitro group on the phenyl ring as compared to the inactivity of its closely related derivative **6** having a *meta* nitro group on phenyl ring proves our hypothesis that a suitable group at a suitable position of the phenyl ring of benzilidine part of molecules is a prerequisite for *β*-glucornidase inhibitory potential in these *N*-acylhydrazone Schiff bases. Comparison of activity of chloro-containing compounds **7** (IC_50_ = 14.7 ± 0.19 µM), **8** (IC_50_ = 15.4 ± 1.56 µM), and **9 ** (inactive) demonstrated that the nature and location of a substitution is important for *β*-glucornidase inhibitory potential. Dichloro-substituted compounds **9** and **10** were found to be completely inactive which further proves our hypothesis. Compound **11** (IC_50_ = 19.6 ± 0.62 µM) having an *ortho* fluoro group showed excellent activity, but a little less than analogous chloro compounds 7 and 8. We also evaluated the effect of heterocyclic ring-containing derivatives, and it was observed that the five membered heterocyclic thiophene ring-containing derivative **12** (IC_50_ 30.7 ± 1.49 µM) produced remarkable activity, while on the other hand five membered heterocyclic rings like furan and its methyl derivatives **13** and **14** were found to be completely inactive. Almost all mono-, di- and trihydroxy substituted compounds **16**, **17**, **18**, **19**, and **20** found to be completely inactive, but unexpectedly compound **15** (IC_50_ = 12.0 ± 0.16 µM) which bears 2,3-dihydroxy substitution, was found to be very efficient and displayed remarkable activity, better than the standard, but, compound **16**, also a 3,4-dihydroxy derivative did not show any activity. *N*-acylhydrazones Schiff base **21** (IC_50_ = 13.7 ± 0.40 µM) synthesized from 1-napthaldehyde was found to be more active than **22** (IC_50_ = 22.0 ± 0.14 µM) which was synthesized from 2-napthaldehyde, both without any substitution. Remaining compounds **23**–**28** were found to be completely inactive. This pattern of activity reveals that the substituent and its position on the phenyl ring of benzylidine part is a driving force for *β*-glucornidase inhibition activity.

In conclusion, a number of potential lead molecules has been identified as *β*-glucuronidase inhibitors. Compounds **1**, **5**, **7**, **8**, **11**, **12**, **15**, **21**, and **22** demonstrated excellent activity and it is anticipated that by slight synthetic modification in these molecules, some new most active *β*-glucuronidase inhibitors can be developed.

## 3. Experimental

### 3.1. General Information

^1^H-NMR experiments were performed on an Avance-Bruker AM 300 MHz instrument (Wissembourg Cedex, France). A Carlo Erba Strumentazione-Mod-1106 (Milan, Italy) used to measure CHN analysis. EI MS was performed on a Finnigan MAT-311A (Bremen, Germany). Thin layer chromatography (TLC) was carried out on pre-coated silica gel glass plates (Kieselgel 60, 254, E. Merck, Darmstadt, Germany). The chromatograms were visualized by UV at 254 and 365 nm or iodine vapours.

### 3.2. Biological Assays

*β*-Glucuronidase (E.C. 3.2.1.31 from bovine liver, G-0251) and *p*-nitrophenyl-*β*-D-glucuronide (N-1627) were purchased From Sigma Chemical Co. (St. Louis, MO, USA). Anhydrous Na_2_CO_3_ and all other reagents of standard grade were obtained from E. Merck. The anhydrous EtOH and CHCl_3_ used in the experiments were dried employing the standard methods. All other solvents and reagents like the benzoyl chloride were of standard grade.

### 3.3. Assay for β-D-Glucuronidase

*β*-D-Glucuronidase inhibition was determined by measuring the absorbance of the *p*-nitrophenol which is produce from the substrate at 405 nm. The total reaction volume was 250 µL. The reaction mixture contains 5 µL of test compound solution, 185 µL of 0.1 M acetate buffer, and 10 µL of enzyme, and it was incubated at 37 °C for 30 min. The plates were read on a multiplate reader at 405 nm after the addition of 50 µL of 0.4 mM *p*-nitrophenyl-*β*-D-glucuronide. All assays were performed in triplicate.

### 3.4. Typical Method for the Synthesis of Compounds **1**–**28**

To a mixture of 2-(4-chloro-2-methylphenoxyacetic acid) hydrazide (1 mmol) in methanol (25 mL) was added a substituted aldehyde (1 mmol) and 3 drops of glacial acetic acid and the mixture was refluxed for 3 h. After completion of the reaction (TLC analysis), it was cooled and evaporated on a rotary evaporator. The resultant crude product was crystallized from methanol to afford 80%–90% yields of pure product. The structures of synthetic compounds **1**–**28** were determined by different spectroscopic techniques, including ^1^H-NMR, and EI MS spectroscopy.

*2-(4-Chloro-2-methylphenoxy)-N'-[(3-(2-methoxyphenyl)-2-propenylidene]acetohydrazide* (**1**). Yield: 68%; ^1^H-NMR (DMSO-*d_6_*) *δ*: 8.04 (d, 1H, *J* = 9.3 Hz, N=CH-CH), 7.63 (dd, 1H, *J*_5',6'_ = 7.8 Hz, *J*_5',3'_ = 1.2 Hz, H-6'), 7.34 (dd, 1H, *J*_CH-HC=CH_ = 8.1 Hz, CH-HC=CH), 7.23 (d, 1H, *J*_HC=CH_ = 6 Hz, HC=CH), 7.12–7.20 (m, 2H, H-3/5) 6.85–7.06 (m, 3H, H- 3'/4'/5'), 6.80 (d, 1H, *J*_6,5_ = 9 Hz, H-6), 5.17 (s, 2H, -OCH_2_), 3.84 (s, 3H, -OCH_3_), 2.23 (s, 3H, CH_3_); EI MS: *m/z* (%) 358 (M^+^, 30), 327 (100),189 (40), 175 (55), 159 (100), 125 (75.0), Anal. Calcd for C_19_H_19_ClN_2_O_3_, C = 63.60, H = 5.34, N = 7.81. Found: C = 63.55, H = 5.31, N = 7.80.

*2-(4-Chloro-2-methylphenoxy)-N'-[(3-(4-methoxyphenyl)-2-propenylidene]acetohydrazide* (**2**). Yield: 70%; ^1^H-NMR (DMSO-*d_6_*) *δ*: 8.02 (d, 1H, *J* = 9.3Hz, N=CH-CH), 7.55 (d, 2H, *J*_2',3'_ = *J*_6',5'_ = 8.7 Hz, H-2'/6'),7.12–7.23 (m, 2H, H-3/5),7.01 (d, 2H, *J*_3',2'_* = J*_5',6'_ = 7.0 Hz, H-3'/5'), 6.76–6.87 (m, 2H, HC = CH), 6.92 (d, 1H, *J*_5,6_ = 8.4 Hz, H-6), 5.03 (s, 2H, -OCH_2_), 3.76 (s, 3H, OCH_3_), 2.21 (s, 3H, CH_3_); *m/z* (%) 358 (M^+^, 95), 189 (40), 175 (85), 159 (100), 125 (60). Anal. Calcd for C_19_H_19_ClN_2_O_3_, C = 63.60, H = 5.34, N = 7.81. Found: C = 63.57, H = 5.32, N = 7.79.

*2-(4-Chloro-2-methylphenoxy)-N'-[(4-ethoxyphenyl)methylidene]acetohydrazide* (**3**). Yield: 80%; ^1^H-NMR (DMSO-*d_6_*) *δ*: 8.20 (s, 1H, -N=CH), 7.62 (d, 2H, *J*_2',3'_* = J*_6',5'_ = 8.7 Hz, H-2'/6'), 7.23–7.12 (m, 2H, H-3/5), 6.84 (d, 2H, *J*_3',2'_* = J*_5',6'_ = 8.7 Hz, H-3'/5'), 6.84 (d, 1H, *J_6,5_* = 8.7 Hz, H-6), 5.13 (s, 2H, OCH_2_), 4.04 (q, 2H, *J* = 6.9 Hz, -CH_2_), 2.22 (s, 3H, -CH_3_), 1.32 (t, 3H, *J* = 6.9 Hz, -CH_3_); EI MS: *m/z* (%) 347 (M^+^, 56), 205 (95), 155 (89), 147 (92), 119 (73). Anal. Calcd for C_18_H_19_ClN_2_O_3_, C = 62.34, H = 5.52, N = 8.08. Found: C = 62.33, H = 5.50, N = 8.03.

*2-(4-Chloro-2-methylphenoxy)-N'-[(3,4,5-trimethoxyphenyl) methylidene]acetohydrazide* (**4**). Yield: 72%; ^1^H-NMR (DMSO-*d_6_*) *δ*: 8.43 (s, 1H, -N=CH), 7.56 (d, 1H, *J*_2',6'_ = 2.7 Hz, H-2'), 7.23 (m, 1H, H-5), 7.13 (d, 1H, *J*_3,5_ = 2.7 Hz, H-3), 6.92(s, 1H, H-6'), 6.83 (d, 1H, *J*_5,6_ = 8.7 Hz H-6), 5.13 (s, 2H, -OCH_2_), 3.82 (s, 9H, -OCH_3_), 2.22 (s, 3H, -CH_3_); EI MS: *m/z* (%) 392 (M^+^, 62.1), 251 (15.5), 193 (100), 179 (88.9), 155 (26.8). Anal. Calcd for C_19_H_21_ClN_2_O_5_, C = 58.09, H = 5.39, N = 7.13. Found: C = 58.05, H = 5.37, N = 7.11.

*2-(4-Chloro-2-methylphenoxy)-N'-[(2-nitrophenyl)methylidene]acetohydrazide* (**5**). Yield: 65%; ^1^H-NMR (DMSO-*d_6_*) *δ*: 8.09(s, 1H, N=CH), 8.29 (m, 2H, H-4'/5'), 7.98 (d, 2H, *J*_3',4'_ = *J*_6',5'_ = 9, H-3'/6') 7.24 (m, 1H, H-5), 7.13 (d, 1H, *J*_3,5_ = 2.7 Hz, H-3), 6.89 (d, 1H, *J*_6,5_ = 8.7, H-6), 5.22 (s, 2H, -OCH_2_), 2.22 (s, 3H, -CH_3_); EI MS: *m/z* (%) 347 (M^+^, 72), 206 (100), 155 (83.3), 125 (75.8). Anal. Calcd for C_16_H_14_ClN_3_O_4_, C = 55.26, H = 4.06, N = 12.08. Found: C = 55.23, H = 4.02, N = 12.04.

*2-(4-Chloro-2-methylphenoxy)-N'-[(3-nitrophenyl)methylidene]acetohydrazide* (**6**). Yield: 70%; ^1^H-NMR (DMSO-*d_6_*) *δ*: 8.40 (s, 1H, -N=CH), 8.51 (d,1H, *J*_5',6'_ = 7.2 Hz H-6'), 8.12–8.25 (m, 2H, H-2'/5'),7.76 (m, 1H, H-4'), 7.12-7.24 (m, 2H, H-3/5), 6.90 (dd, 1H, *J*_6,5_ = 6.2 Hz, H-6),5.22 (s, 2H, OCH_2_), 2.23 (s, 3H, CH_3_); EI MS: *m/z* (%) 347 (M^+^, 92), 206 (100), 178 (28.4), 141 (17.5), 125 (39.9). Anal. Calcd for C_19_H_21_ClN_2_O_5_, C = 58.09, H = 5.39, N = 7.13. Found: C = 58.06, H = 5.37, N = 7.12.

*2-(4-Chloro-2-methylphenoxy)-N'-[-(4-chlorophenyl)methylidene]acetohydrazide* (**7**). Yield: 63%; ^1^H-NMR (DMSO-*d_6_*) *δ*: 8.27 (s, 1H, -N=CH), 7.73 (d, 2H, *J*_2',3'_ = *J*_6',5'_ = 8.7 Hz, H-2'/6'), 7.51 (d, 2H, *J*_3',2'_* = J*_5',6'_ = 8.1 Hz, H-3'/5'), 7.13–7.23 (m, 2H, H-3/5), 6.89 (d, *J*_6,5_ = 7.8 Hz, 1H, H-6), 5.15 (s, 2H, -OCH_2_), 2.19 (s, 3H, CH_3_); EI MS: *m/z* (%) 336 (M^+^, 20), 195 (35), 155 (55), 125 (80), 89 (100). Anal. Calcd for C_16_H_14_Cl_2_N_2_O_2_, C = 56.99, H = 4.18, N = 8.31. Found: C = 56.95, H = 4.15, N = 8.28.

*2-(4-Chloro-2-methylphenoxy)-N'-[-(2-chlorophenyl)methylidene]acetohydrazide* (**8**). Yield: 66%; ^1^H-NMR (DMSO-*d_6_*) *δ*: 8.26 (s,1H, -N=CH), 7.77 (m, 2H, H-3'/6'), 7.48 (br.t, 2H, *J*_4',5'_* = J*_5',4'_ = 8.5 Hz, H-4'/5'), 7.13–7.24 (m, 1H, H-3/5), 6.89 (d, 1H, *J*_6,5_ = 8.7 Hz, H-6), 5.15 (s, 2H, -OCH_2_), 2.23 (s, 3H, -CH_3_), 2.19 (s, 3H, CH_3_); EI MS: *m/z* (%) 336 (M^+^, 10), 195 (30), 155 (40), 125 (75), 89 (100). Anal. Calcd for C_16_H_14_Cl_2_N_2_O_2_, C = 56.99, H = 4.18, N = 8.31. Found: C = 56.96, H = 4.15, N = 8.27.

*2-(4-Chloro-2-methylphenoxy)-N'-[(3,4-dichlorophenyl)methylidene]acetohydrazide* (**9**). Yield: 70%; ^1^H-NMR (DMSO-*d_6_*) *δ*: 8.25 (s, 1H, =N-CH), 7.96 (s, 1H, H-2'), 7.68 (br. s, 2H, 5'/6'), 7.12–7.23 (m, *2*H, H-3/5), 6.88 (d, 1H, *J*_6,5_ = 7.0 Hz H-6), 5.08 (s, 2H, OCH_2_), 2.22 (s, 3H, CH_3_); EI MS: *m/z* (%) 371 (M^+^, 92.2), 228 (100), 155 (87.1), 125 (89.8). Anal. Calcd for C_16_H_13_Cl_3_N_2_O_2_, C = 51.71, H = 3.53, N = 7.54. Found: C = 51.68, H = 3.50, N = 7.52.

*2-(4-Chloro-2-methylphenoxy)-N'-[(2, 6-dichlorophenyl)methylidene]acetohydrazide* (**10**). Yield: 60%; ^1^H-NMR (DMSO-*d_6_*) *δ*: 8.48 (s,1H, -N=CH), 7.57 (d, 2H, *J*_3',4'_* = J*_5',4'_ = 8.7 Hz, H-3'/5'), 7.44 (dd, 1H, *J*_4,5_ = 7.2 Hz, H-4'), 7.24–7.11 (m, 2H, H-3/5), 6.78 (d, 1H, *J*_6,5_ = 8.7 Hz, H-6), 5.09 (s, 2H, -OCH_2_), 2.18 (s, 3H, CH_3_); EI MS: *m/z* (%) 370 (M^+^, 17.3), 229 (39.5), 155 (60.1), 125 (100), 89 (62). Anal. Calcd for C_16_H_13_Cl_3_N_2_O_2_, C = 51.71, H = 3.53, N = 7.54. Found: C = 51.66, H = 3.51, N = 7.52.

*2-(4-Chloro-2-methylphenoxy)-N'-[-(2-fluorophenyl)methylidene]acetohydrazide* (**11**). Yield: 74%; ^1^H-NMR (DMSO-*d_6_*) *δ*: 8.52 (s, 1H, -N=CH), 7.95 (dd, 1H, *J*_4',5'_ = 8.1 H-5') 7.48 (dd, 1H, *J*_6',5'_ = 6.9 Hz, H-6'), 7.25-7.31 (m, 2H, H-3'/4'), 7.12–7.21 (m, 2H, H-3/5), 6.90 (d, 1H, *J*_6,5_ = 8.7 Hz, H-6), 5.17 (s, 2H, -OCH_2_), 2.23 (s, 3H, CH_3_); EI MS: *m/z* (%) 320 (M^+^, 12), 179 (80), 125 (100), 89 (75.8). Anal. Calcd for C_16_H_14_ClFN_2_O_2_, C = 59.91, H = 4.40, N = 8.73. Found: C = 59.89, H = 4.39, N = 8.72.

*2-(4-Chloro-2-methylphenoxy)-N'-[3-thienylmethylidene]acetohydrazide* (**12**). Yield: 68%; ^1^H-NMR (DMSO-*d_6_*) *δ*: 8.07 (s, 1H, -N=CH), 7.12–7.24 (m, 3H, H-3, 5, 6), 6.73–9.92 (m, 3H, H-2'/4'/5'), 5.10 (s, 2H, -OCH_2_), 2.22 (s, 3H, CH_3_); EI MS: *m/z* (%) 308 (M^+^, 15), 199 (10), 155 (45), 125 (100). Anal. Calcd for C_14_H_13_ClN_2_O_2_S, C = 54.46, H = 4.24, N = 9.07. Found: C = 54.45, H = 4.22, N = 9.04.

*2-(4-Chloro-2-methylphenoxy)-N'-[-2-furylmethylidene]acetohydrazide* (**13**). Yield: 70%; ^1^H-NMR (DMSO-*d_6_*) *δ*: 8.17 (s, 1H, -N=CH), 7.11–7.23 (m, 2H, H-3,5), 6.90 (d, 2H, *J*_3',4'_= *J*_5',4'_ = 3.3 Hz, H-3'/5'), 6.81(d, 1H, *J*_6,5_ = 8.7 Hz, 6), 6.62 (dd, 1H, *J*_4,3_*= J*_4,5_ = 3.3 Hz, 4'), 5.07 (s, 2H, -OCH2), 2.22 (s, 3H, -CH3); EI MS: *m/z* (%) 292 (M+, 35), 155 (50), 151 (80.), 125 (100). Anal. Calcd for C_14_H_13_ClN_2_O_3_, C = 57.44, H = 4.48, N = 9.57. Found: C = 57.44, H = 4.46, N = 9.56.

*2-(4-Chloro-2-methylphenoxy)-N'-[-(5-methyl-2-furyl)methylidene]acetohydrazide* (**14**). Yield: 72%; ^1^H-NMR (DMSO-*d_6_*) *δ:* 8.07 (s, 1H, -N=CH), 7.11–7.23 (m, 2H, H-3,5), 6.88 (d, 1H, *J*_5,6_ = 8.7 Hz, H-6), 6.80 (d, 1H, *J*_3',4'_ = 5.4 Hz, 3'), 6.24 (d, 1H, *J*_4',3'_ = 2.7 Hz, 4'), 5.07 (s, 2H, -OCH_2_), 2.21 (s, 3H, -CH_3_) 2.19 (s, 3H, -CH_3_); EI MS: *m/z* (%) 306 (M^+^, 20), 65 (30), 137 (100), 125 (40). Anal. Calcd for C_15_H_15_ClN_2_O_3_, C = 58.73, H = 4.93, N = 9.13. Found: C = 58.70, H = 4.96, N = 9.10.

*2-(4-Chloro-2-methylphenoxy)-N'-[-(2,3-dihydroxyphenyl)methylidene]acetohydrazide* (**15**). Yield: 76%; ^1^H-NMR (DMSO-*d_6_*) *δ*: 8.41 (s,1H, -N=CH), 7.24 (m, 1H, H-5), 7.13 (d, 1H, *J*_3,5_ = 2.7 Hz, H-3), 6.89 (d, 1H, *J*_5',6'_ = 8.7 Hz, H-6'), 6.83 (d, 1H, *J*_6,5_ = 8.7 Hz, H-6), 6.71 (m, 2H, H-3'/4'), 5.15 (s, 2H, -OCH_2_), 2.19 (s, 3H, -CH_3_); EI MS: *m/z* (%) 334 (M^+^, 100),300 (9), 175 (89.7), 193 (35), 179 (50), 137 (65), 125 (60). Anal. Calcd for C_16_H_15_ClN_2_O_4_, C = 57.41, H = 4.5, N = 8.37. Found: C = 57.40, H = 3.50, N = 8.36.

*2-(4-Chloro-2-methylphenoxy)-N'-[-(3,4-dihydroxyphenyl)methylidene]acetohydrazide* (**16**). Yield: 68%; ^1^H-NMR (DMSO-*d_6_*) *δ*: 8.06 (s, 1H, -N=CH), 7.12-7.19 (m, 2H, H-3/5),7.24 (d, 1H, *J*_2',6'_ = 2.7 Hz, H-2'), 6.85–6.92 (m, 2H ,H-5'/6'), 6.76 (d, *J*_6,5_ = 8.1 Hz, 1H, H-6), 5.10 (s, 2H, -OCH_2_), 2.22 (s, 3H, CH_3_); EI MS: *m/z* (%) 334 (M, 36.8), 193 (23.9), 155 (71.6), 125 (88.8), 77 (100). Anal. Calcd for C_16_H_15_ClN_2_O_4_, C = 57.41, H = 4.5, N = 8.37. Found: C = 57.39, H = 3.50, N = 8.37.

*2-(4-Chloro-2-methylphenoxy)-N'-[1-(4-hydroxyphenyl)ethylidene]acetohydrazide* (**17**). Yield: 70%;^1^H-NMR (DMSO-*d_6_*) *δ*: 7.65 (d, 2H, *J*_2',3'_* = J*_6',5'_ = 8.7 Hz , H-2'/6'), 7.23 (m, 1H, Hz H-6), 7.12 (d, 1H, *J* = 2.4 Hz, H-3), 6.90 (d, *J*_3',2'_* = J*_5',6'_ = 8.7 Hz, 2H, H-3'/5'), 6.77 (d, 1H, *J*_6,5_ = 8.7 Hz, H-6), 5.16 (s, 2H, -OCH_2_), 2.19 (s, 6H, CH_3_); EI MS: *m/z* (%) 332 (M^+^, 56.7), 191 (100), 177 (24.5), 149 (34.5), 134 (37.9). Anal. Calcd for C_17_H_16_ClN_2_O_3_, C = 61.36, H = 5.15, N = 8.42. Found: C = 61.34, H = 5.14, N = 8.40.

*2-(4-Chloro-2-methylphenoxy)-N'-[(3-hydroxyphenyl)methylidene]acetohydrazide* (**18**). Yield: 61%; ^1^H-NMR (DMSO-*d_6_*) *δ*: 8.18 (s, 1H, -N=CH), 7.04-7.24 (m, 5H, Ar-H), 6.79–6.89 (m, 2H, H-5-6), 5.14 (s, 2H, -OCH_2_), 2.22 (s, 3H,-CH_3_); EI MS: *m/z* (%) 318 (M^+^, 97.6), 177 (100), 155 (71), 125 (65.3). Anal. Calcd for C_16_H_15_ClN_2_O_3_, C = 60.29, H = 4.74, N = 8.79. Found: C = 69.27, H = 3.49, N = 8.34.

*2-(4-Chloro-2-methylphenoxy)-N'-[1-(2,4,6-trihydroxyphenyl)ethylidene]acetohydrazide* (**19**). Yield: 62%; ^1^H-NMR (DMSO-*d_6_*) *δ*: 8.16 (s, 1H, -N=CH), 7.21 (br.s, 2H, H-3'/5'), 7.16–7.23 (m, 2H, H-3/5), 6.86 (d, *J*_6,5_ = 8.7 Hz, 1H, H-6), 4.98 (s, 2H, -OCH_2_), 2.18 (s, 3H, CH_3_), 1.93 (s, 3H, CH_3_); EI MS: *m/z* (%) 254 (M^+^, 100), 155 (91.2), 142 (75.5), 125 (100), 113 (100), 99 (86.6).

*2-(4-Chloro-2-methylphenoxy)-N'-[(4-hydroxyphenyl)methylidene]acetohydrazide* (**20**). Yield: 72%; ^1^H-NMR (DMSO-*d_6_*) *δ*: 7.88 (s, 1H, -N=CH), 7.52 (d, 2H, d, 2H, *J*_2',3'_* = J*_6',5'_ = 8.7 Hz , H-2'/6'), 7.24 (d, 1H, *J*_5,6_ = 8.1 Hz, H-5), 7.13(d, 1H, *J* = 2.4 Hz, H-3), 6.88 (d, 2H, *J*_3',2*'*_* = J_5',6'_* = 8.7 Hz, H-3'/5'), 6.79 (d, 1H, *J_6,5_* = 8.7 Hz, H-6), 5.11 (s, 2H, -OCH_2_), 2.22 (s, 3H, CH_3_); EI MS: *m/z* (%) 318 (M^+^, 9.30), 199 (10.78), 177 (27.43), 155 (44), 125 (67.50), 77 (100). Anal. Calcd for C_16_H_15_ClN_2_O_3_, C = 60.29, H = 4.74, N = 8.79. Found: C = 69.26, H = 3.50, N = 8.34.

*2-(4-Chloro-2-methylphenoxy)-N'-[-1-naphthylmethylidene]acetohydrazide* (**21**). Yield: 68%; ^1^H-NMR (DMSO-*d_6_*) *δ*: 8.66 (s, 1H, N=CH), 8.83 (d, *J* = 8.1 Hz 1H, H-8'), 8.61 (d, 1H, *J*_4',3'_ = 8.4 Hz, H-4'), 8.02 (br, d, 2H, *J* = 7.8 Hz, H-2'/5'), 7.93 (d, 1H, *J*_3',4'_ = 7.8 Hz, H-3') 7.65 (m, 2H, H-6'/7'), 7.14–7.25 (m,2H, H-3/5), 6.95 (d, *J*_6,5_ = 8.4 Hz, 1H, H-6), 5.24 (s, 2H, -OCH_2_), 2.26 (s, 3H, -CH_3_); EI MS: *m/z* (%) 352 (M^+^, 100), 211(65), 199. Anal. Calcd for C_20_H_17_ClN_2_O_2_, C = 68.09, H = 4.86, N = 7.94. Found: C = 68.06, H = 4.84, N = 7.92.

*2-(4-Chloro-2-methylphenoxy)-N'-[-2-naphthylmethylidene]acetohydrazide* (**22**). Yield: 70%; ^1^H-NMR (DMSO-*d_6_*) *δ*: 8.43 (s, 1H, N=CH), 8.16 (t, 2H, *J* = 8.4 Hz, H-3',6'), 7.94 (br, s, 2H, H-5/4') 7.56 (t, 2H, *J*_5',6'_ = *J*_8',7'_ = 9.6 Hz, H-5'/8'), 7.22 (s, 1H, H-1'), 7.17 (d, 1H, *J*_7',8'_ = 8.7 Hz, H-7'), 6.92 (m, 2H, H-5/6), 5.23 (s, 2H, OCH_2_), 2.23 (s, 3H, CH_3_); EI MS: *m/z* (%) 352 (M^+^, 72), 211 (60) 199 (35), 169 (45), 153 (100), 127 (60). Anal. Calcd for C_20_H_17_ClN_2_O_2_, C = 68.09, H = 4.86, N = 7.94. Found: C = 68.04, H = 4.85, N = 7.91.

*2-(4-Chloro-2-methylphenoxy)-N'-[(5-Chloro-2-hydroxyphenyl)methylidene]acetohydrazide* (**23**). Yield: 66; ^1^H-NMR (DMSO-*d_6_*) *δ*: 8.23 (s,1H, -N=CH), 7.70 (d, 1H, *J_6',4'_* = 2.7 Hz H-6'),7.12–7.23 (m, 2H, H-3/5), 7.31 (m, 1H, H-4'), 6.93 (d, 1H, *J*_3',4'_ = 7.8Hz, H-3'),6.86 (d, 1H, *J*_6,5_ = 8.7 Hz, H-6), 5.17 (s, 2H, -OCH_2_), 2.22 (s, 3H, -CH_3_); EI MS: *m/z* (%) 352 (M^+^, 83.8), 210 (7.4), 197 (62.6), 155 (100), 141 (46.8). Anal. Calcd for C_16_H_14_Cl_2_N_2_O_3_, C = 54.41, H = 4.00, N = 7.93. Found: C = 54.39, H = 4.00, N = 7.92.

*2-(4-Chloro-2-methylphenoxy)-N'-[1(2,4-dihydroxy-5-nitrophenyl)ethylidene]acetohydrazide* (**24**). Yield: 70%; ^1^H-NMR (DMSO-*d_6_*) *δ*: 8.23 (s, 1H, H-6'), 7.17–7.23 (m, 2H, H-3/5), 6.89 (d, 1H, *J*_6,5_ = 8.7 Hz, H-6), 6.51 (s, 1H, H-3'), 4.82 (s, 2H, -OCH_2_), 2.26 (s, 3H, CH_3_), 2.21 (s, 3H, CH_3_); EI MS: *m/z* (%) 392 (M^+^, 64.4), 251 (72.1), 237 (100), 125 (89.9), 77 (68.5). Anal. Calcd for C_16_H_14_ClN_3_O_6_, C = 50.60, H = 3.72, N = 11.07. Found: C = 50.57, H = 3.70, N = 11.05.

*2-(4-Chloro-2-methylphenoxy)-N'-[(4-hydroxy-3-methoxyphenyl)methylidene]acetohydrazide* (**25**). Yield: 59%; ^1^H-NMR (DMSO-*d_6_*) *δ*: 8.11 (s, 1H, -N=CH), 7.12–7.23 (m, 2H, H-3/5), 7.03 (s, 1H, H-2'), 6.86–6.96 (m, 2H, H-5'/6') 6.83 (d, 1H, *J*_6,5_ = 8.7 Hz, H-6), 5.12 (s, 2H, -OCH_2_), 3.78 (s, 3H, OCH_3_), 2.23 (s, 3H, CH_3_); EI MS: *m/z* (%) 348 (M^+^, 88.5), 207 (51), 193 (100), 155 (43), 125 (46.9). Anal. Calcd for C_17_H_17_ClN_2_O_4_, C = 58.54, H = 4.91, N = 8.03. Found: C = 58.53, H = 4.90, N = 8.02.

*2-(4-Chloro-2-methylphenoxy)-N'-{-[4-(methylsulfanyl)phenyl]methylidene}acetohydrazide* (**26**). Yield: 63%; ^1^H-NMR (DMSO-*d_6_*) *δ*: 8.22 (s, 1H, -N=CH), 7.63 (d, 2H, *J*_2,3_* = J*_6,5_ = 8.4 Hz, H-2'/6'), 7.31 (d, 2H, *J*_3,2_* = J*_5,6_ = 8.4 Hz, H-3'/5'),7.23 (m, 1H, H-5), 7.13 (d, 1H, *J* = 2.7 Hz, H-3), 6.89 (d, *J*_6,5_ = 8.4Hz, 1H, H-6), 5.15 (s, 2H, -OCH_2_), 2.19 (s, 3H, CH_3_); EI MS: *m/z* (%) 348 (M^+^, 60), 207 (25), 149 (100), 125 (35), 118 (35). Anal. Calcd for C_17_H_17_ClN_2_O_2_S, C = 58.53, H = 4.91, N = 8.03. Found: C = 58.51, H = 4.90, N = 8.02.

*2-(4-Chloro-2-methylphenoxy)-N'-[(2-methylphenyl)methylidene]acetohydrazide* (**27**). Yield: 67%; ^1^H-NMR (DMSO-*d_6_*) *δ*: 8.25 (s, 1H, -N=CH), 7.78 (d, *J* = 7.5 Hz, 1H, H-6'), 7.30–7.21 (m, 3H, H-3'/4'/5'), 7.13-7.17 (m, 2H, H-3/5), 6.84 (d, 1H, *J*_6,5_ = 8.7 Hz, H-6), 5.15 (s, 2H, -OCH_2_), 2.23 (s,3H, -CH_3_), 2.19 (s, 3H, CH_3_); EI MS: *m/z* (%) 316 (M^+^, 79.4), 199 (78.9), 175 (89.7), 155 (100), 141 (93.2), 118 (94.2). Anal. Calcd for C_17_H_17_ClN_2_O_2_, C = 64.46, H = 4.41, N = 8.84. Found: C = 64.45, H = 4.40, N = 8.83.

*2-(4-Chloro-2-methylphenoxy)-N'-[(4-methylphenyl)methylidene]acetohydrazide* (**28**). Yield: 63%; ^1^H-NMR (DMSO-*d_6_*) *δ*: 8.23 (s, 1H, -N=CH), 7.59 (d, 2H, *J_2',3'_* = *J_6',5'_* = 7.8 Hz, H-2'/6'), 7.24 (d, 2H, *J*_3',2'_= *J*_5',6'_ = 8.1 Hz, H-3'/5'),7.13 (d, *J* = 2.7 Hz, 1H, H-3), 7.17 (m, 1H, H-5), 6.85 (d, *J*_6,5_ = 8.7 Hz, 1H, H-6), 5.15 (s, 2H, -OCH_2_), 2.32 (s, 3H, CH_3_), 2.19 (s, 3H, CH_3_); EI MS: *m/z* (%) 316 (M^+^, 9.61), 199 (10.80), 175 (36.26), 155 (47.70), 125 (71.62). Anal. Calcd for C_17_H_17_ClN_2_O_2_, C = 64.46, H = 4.41, N = 8.84. Found: C = 64.44, H = 4.40, N = 8.82.

## 4. Conclusions

A number of potential lead molecules **1**, **5**, **7**, **8**, **11**, **12**, **15**, **21**, and **22** have been identified as *β*-glucuronidase inhibitors and it is anticipated that by slight synthetic modification in these molecules, some new most active *β*-glucuronidase inhibitors can be developed.

## Supplementary Materials

Supplementary materials can be accessed at: http://www.mdpi.com/1420-3049/19/7/8788/s1.
