# Supplementary material for: Phenoxyacetohydrazide Schiff Bases: β-Glucuronidase Inhibitors
Source: Molecules. 2014 Jun 25;19(7):8788–802. doi: 10.3390/molecules19078788 (PMC6271590; doi:10.3390/molecules19078788)

Supplementary Materials

Figure S1. 1H-NMR spectrum (300 MHz, DMSO-d_6) of compound **1**.

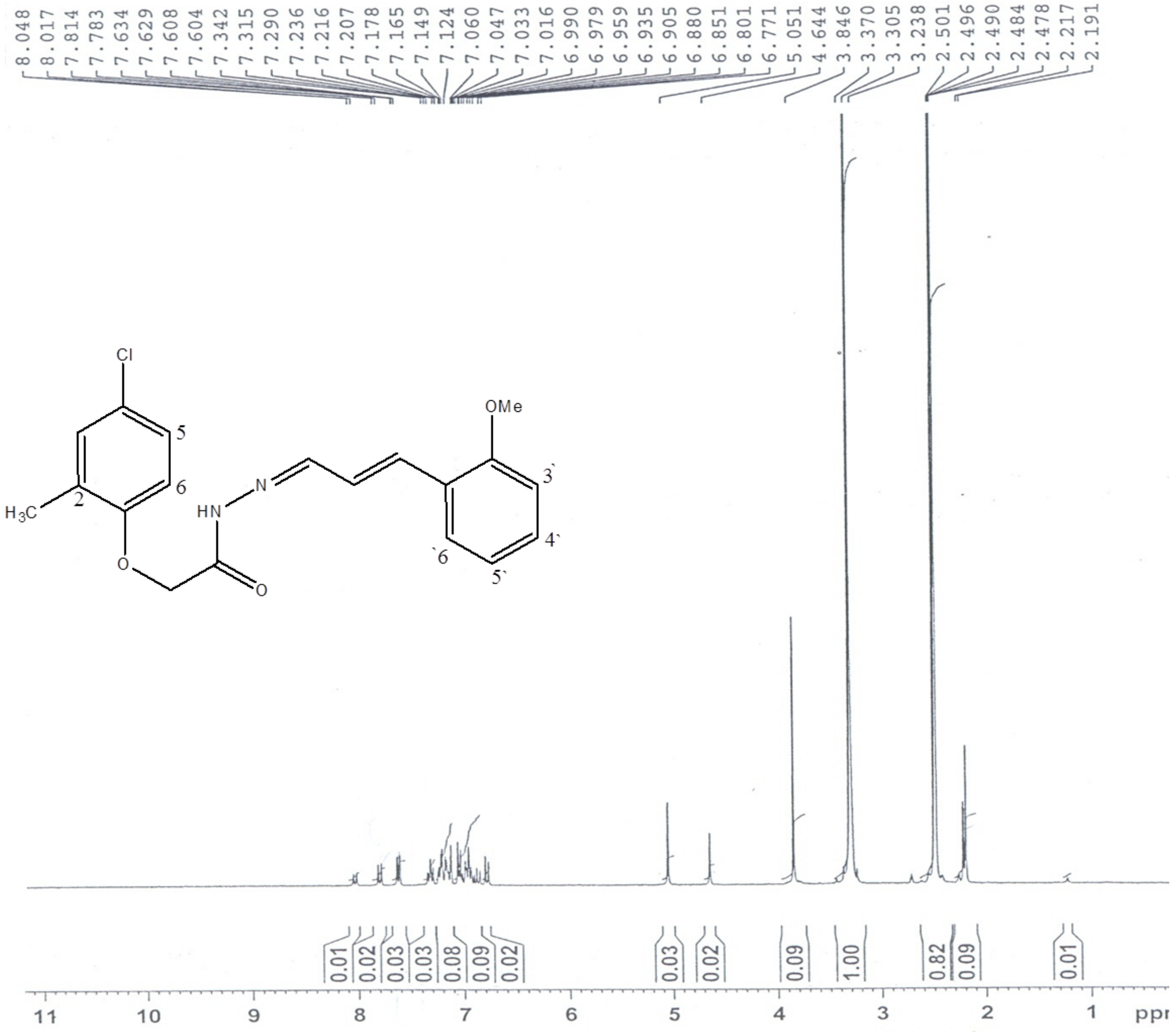

Figure S2. 1H-NMR spectrum (300 MHz, DMSO-d_6) of compound **2**.

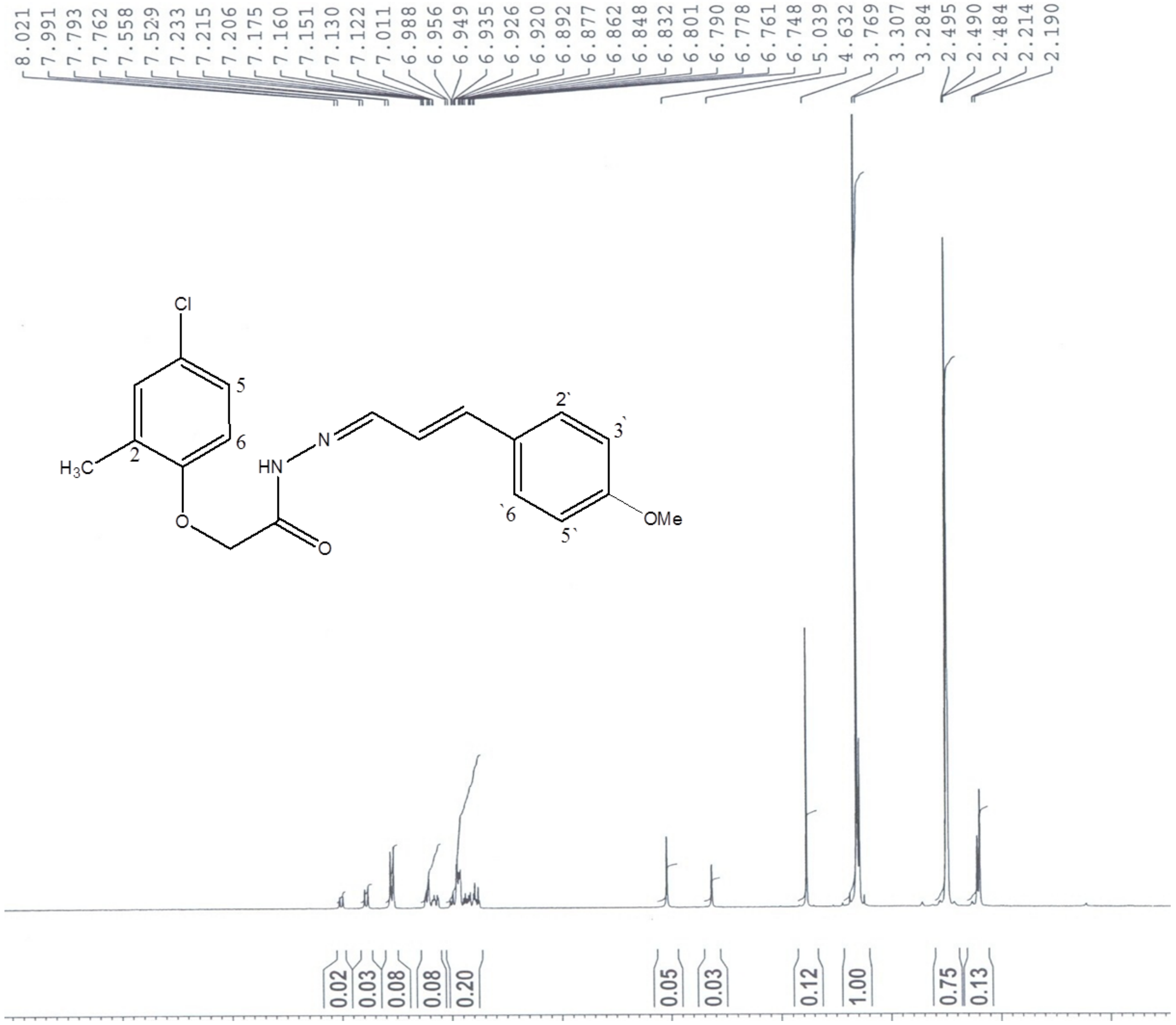

Figure S3. 1H-NMR spectrum (300 MHz, DMSO-d_6) of compound **3**.

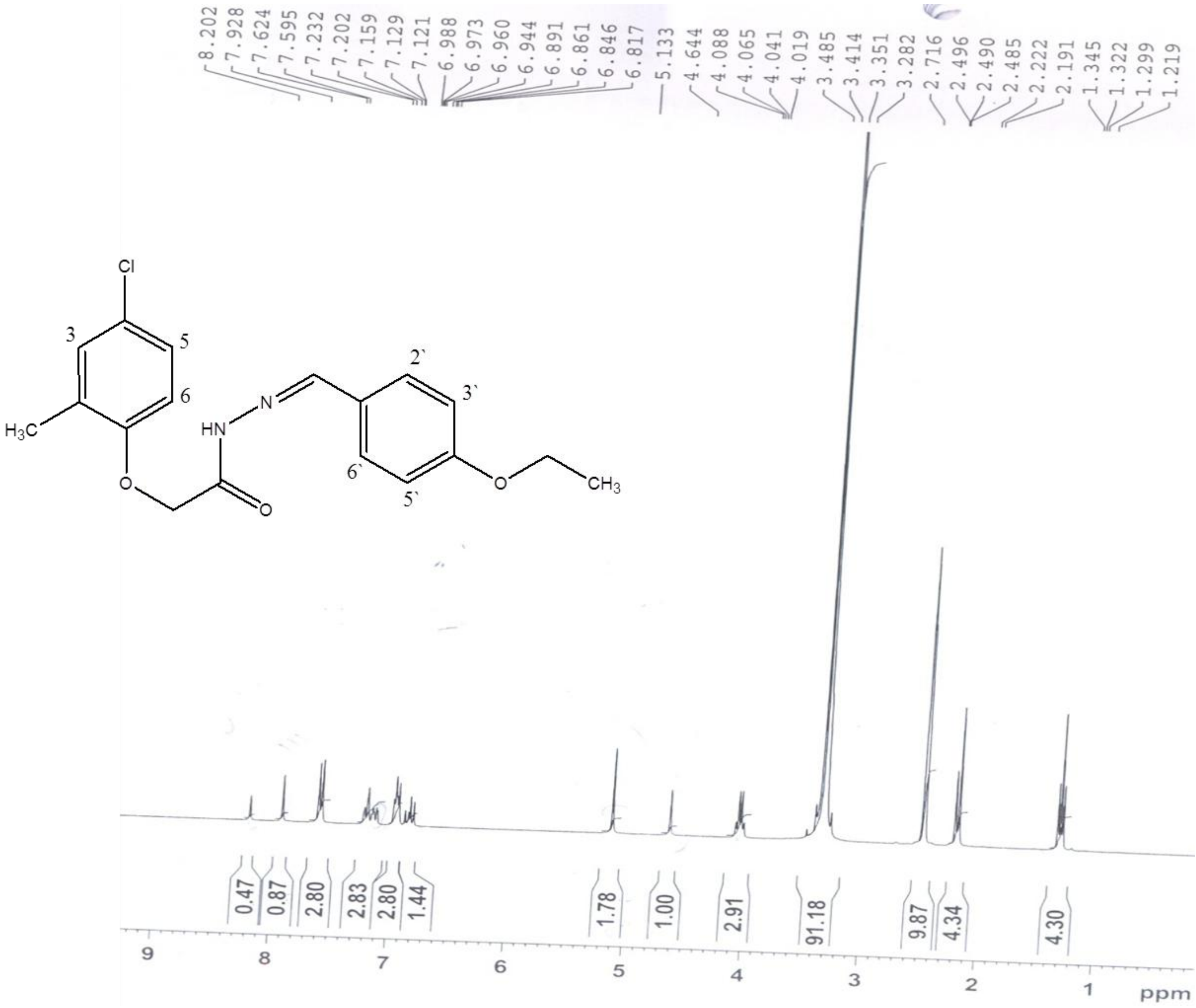

Figure S4. 1H-NMR spectrum (300 MHz, DMSO-d_6) of compound **4**.

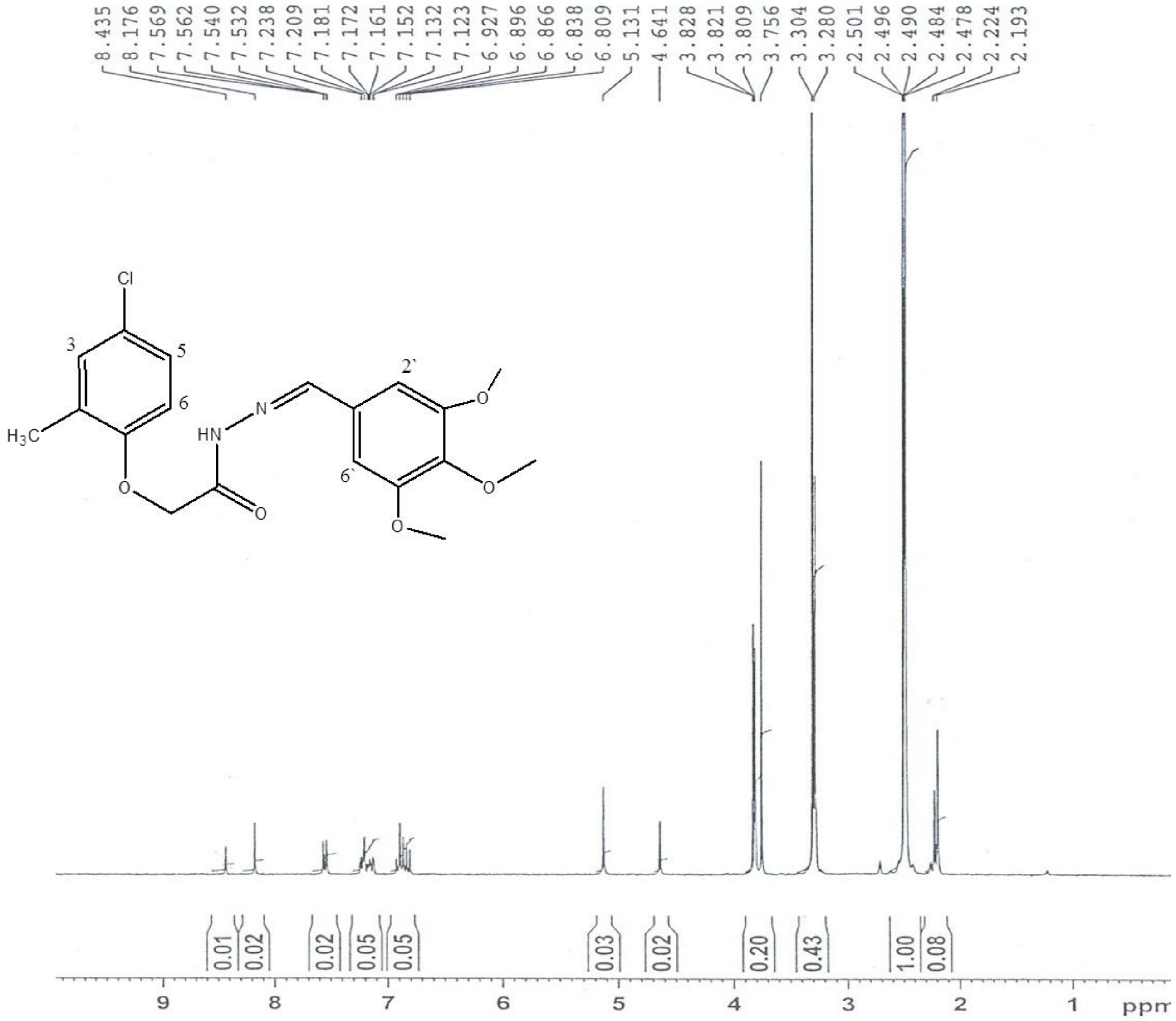

Figure S5. 1H-NMR spectrum (300 MHz, DMSO-d_6) of compound **5**.

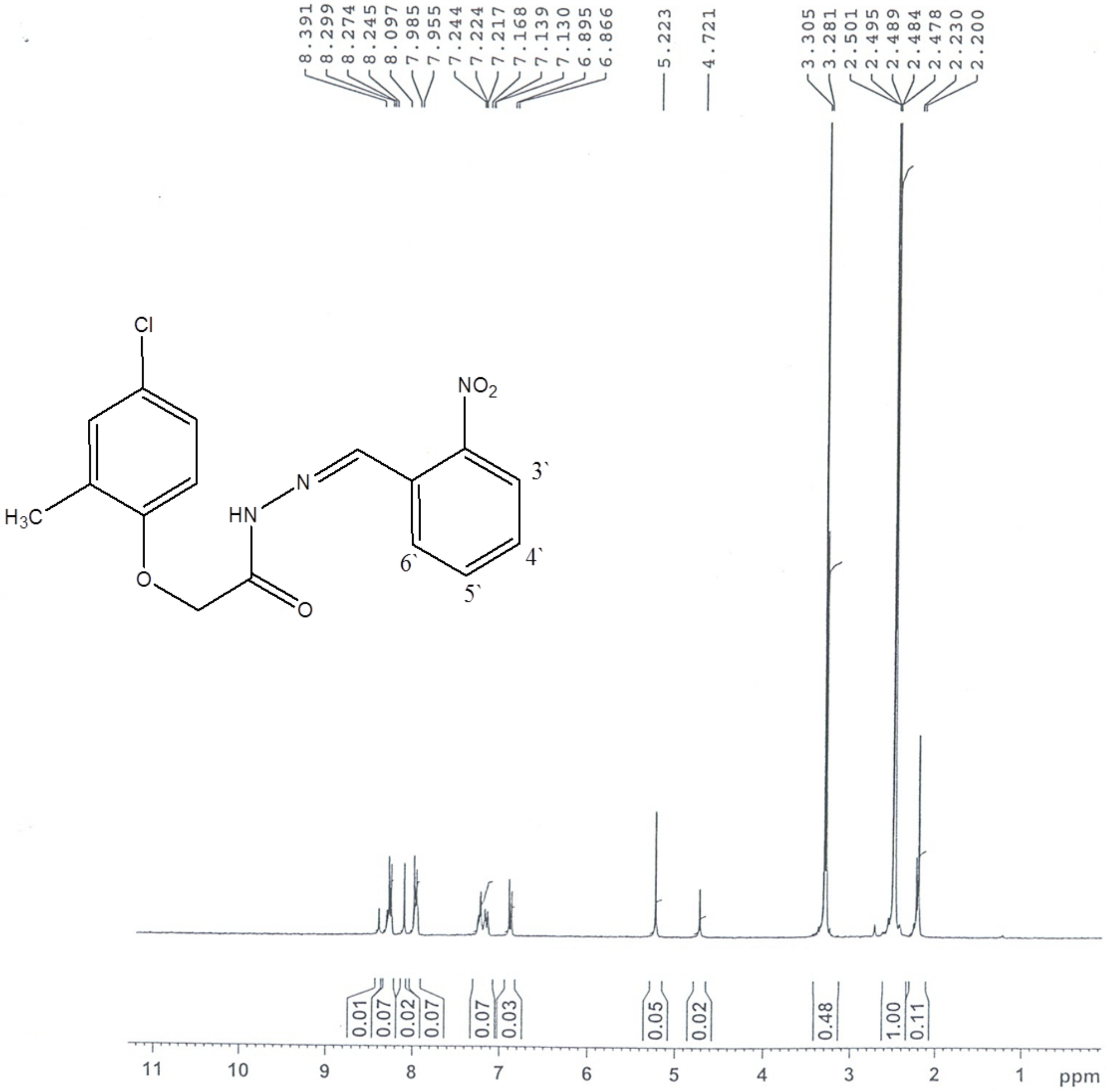

Figure S6. 1H-NMR spectrum (300 MHz, DMSO-d_6) of compound **6**.

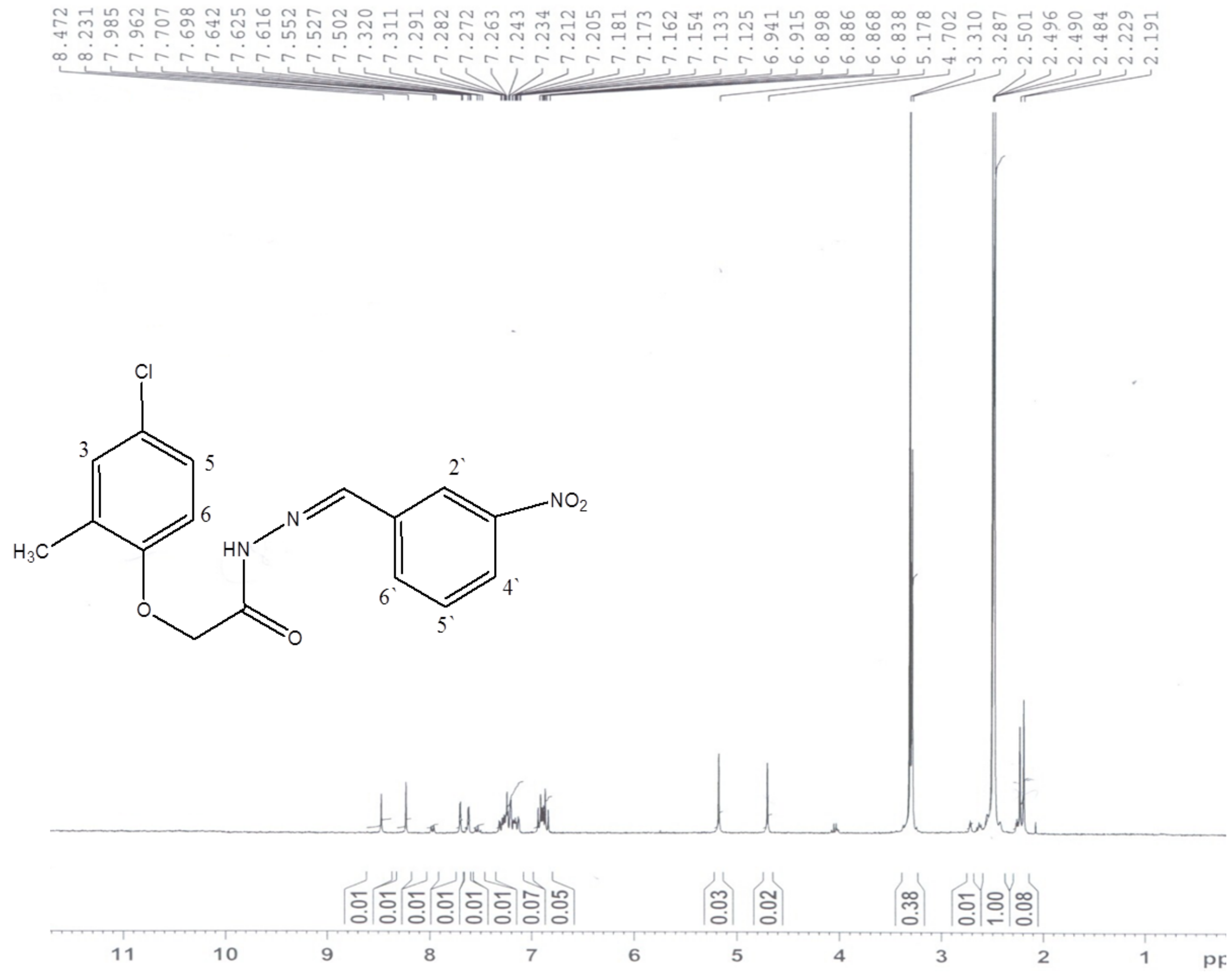

Figure S7. 1H-NMR spectrum (300 MHz, DMSO-d_6) of compound **7**.

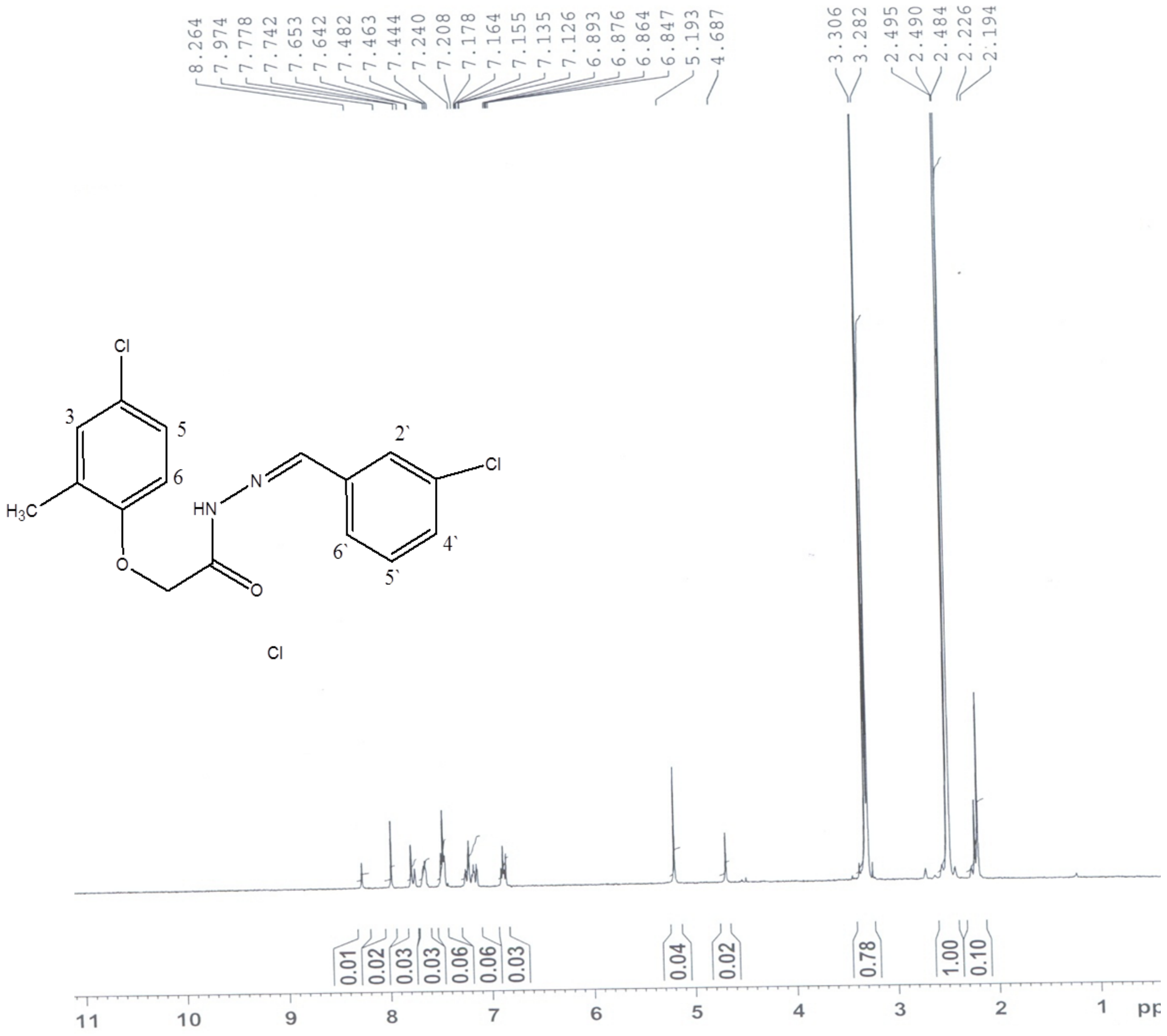

Figure S8. 1H-NMR spectrum (300 MHz, DMSO-d_6) of compound **8**.

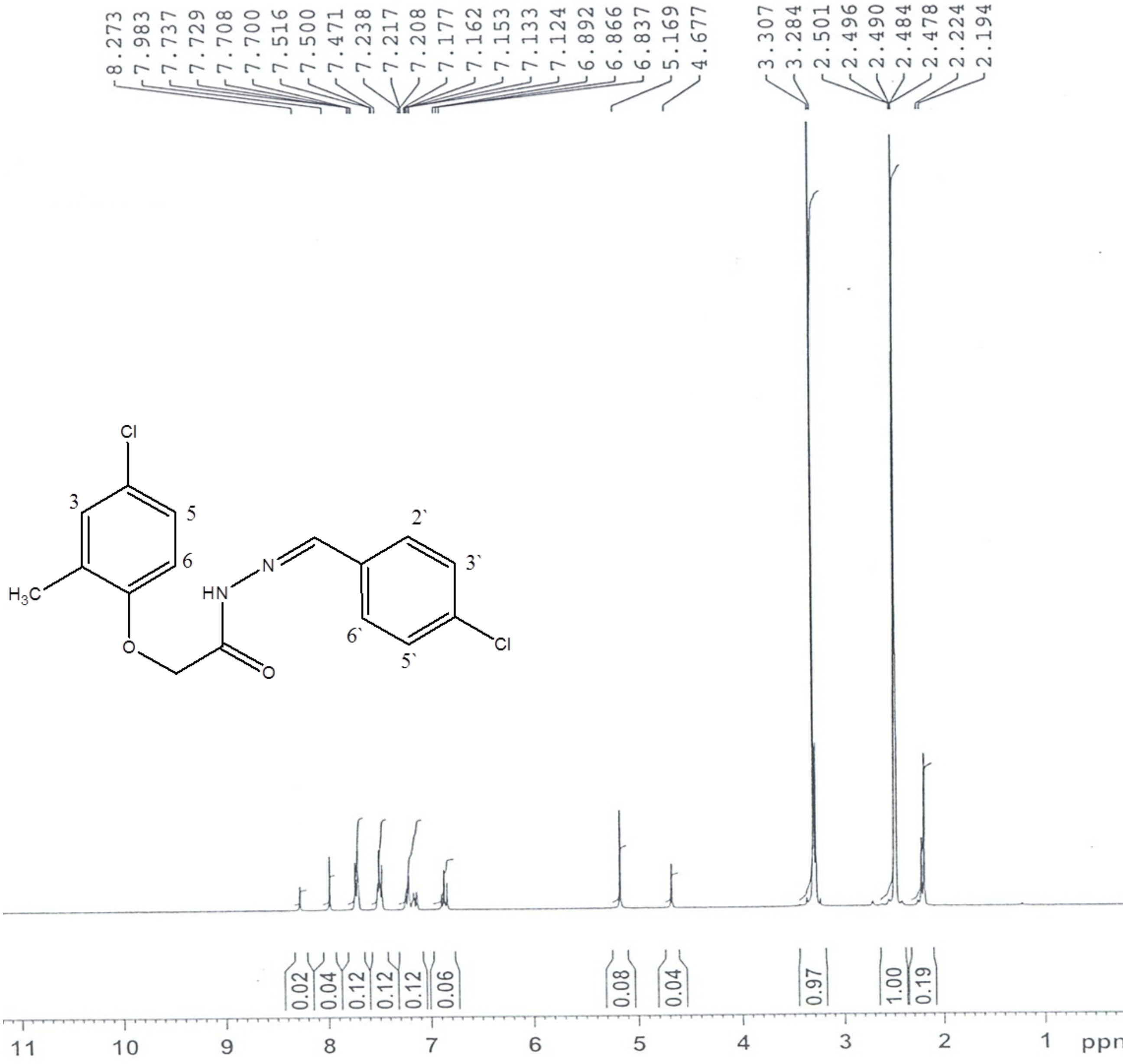

Figure S9. 1H-NMR spectrum (300 MHz, DMSO-d_6) of compound **9**.

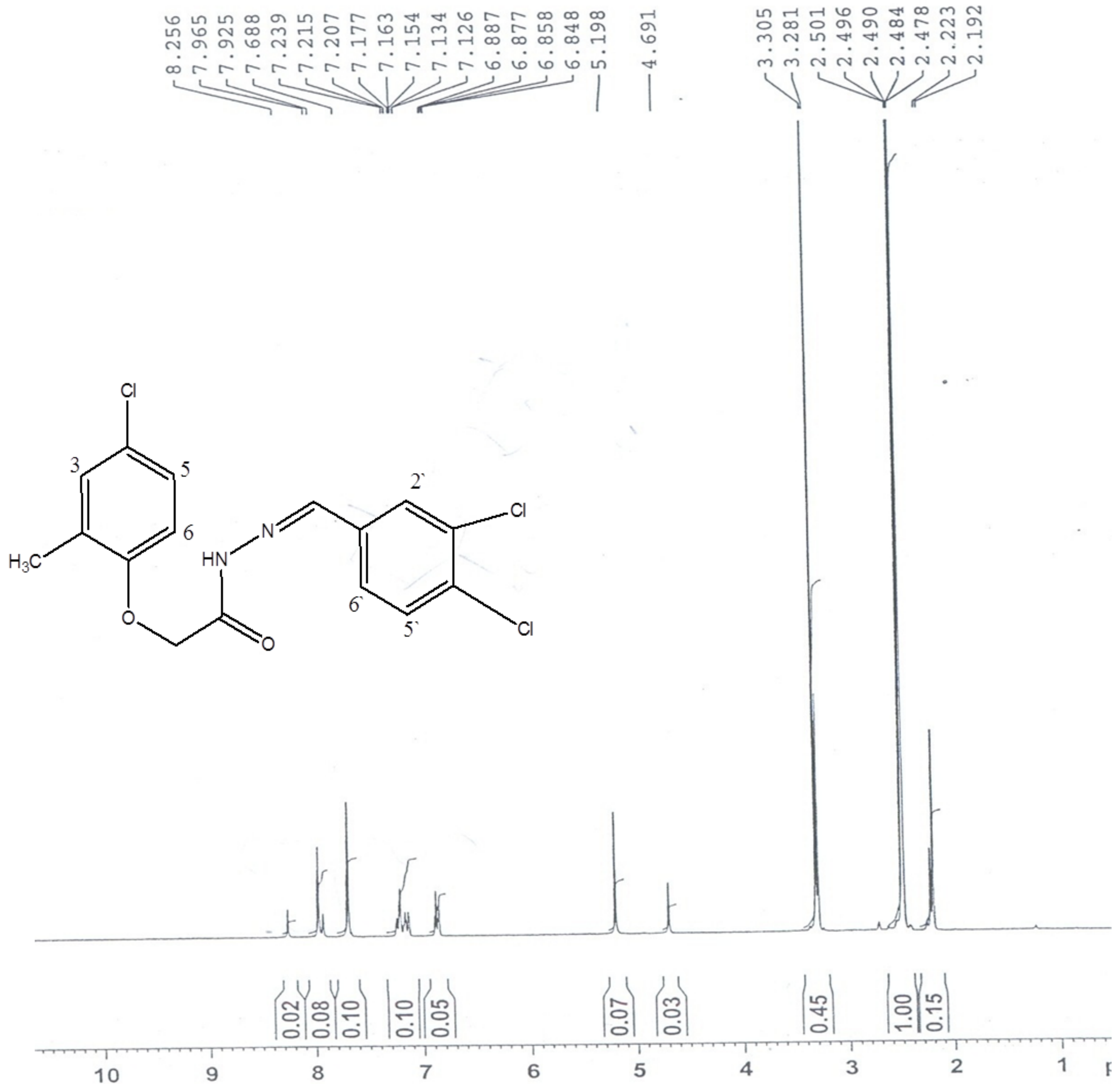

Figure S10. 1H-NMR spectrum (300 MHz, DMSO-d_6) of compound **10**.

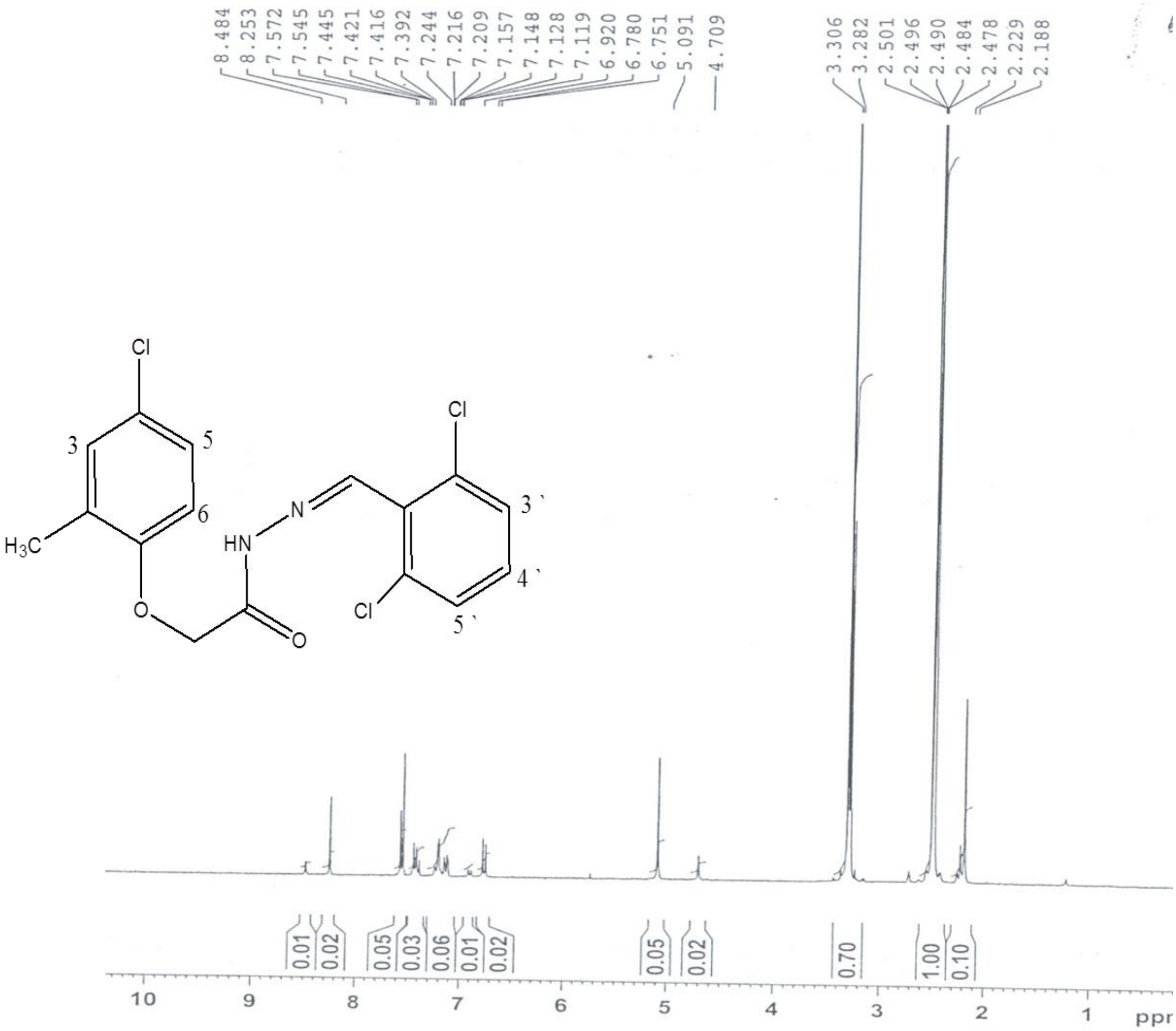

Figure S11. 1H-NMR spectrum (300 MHz, DMSO-d$_6$) of compound **11**.

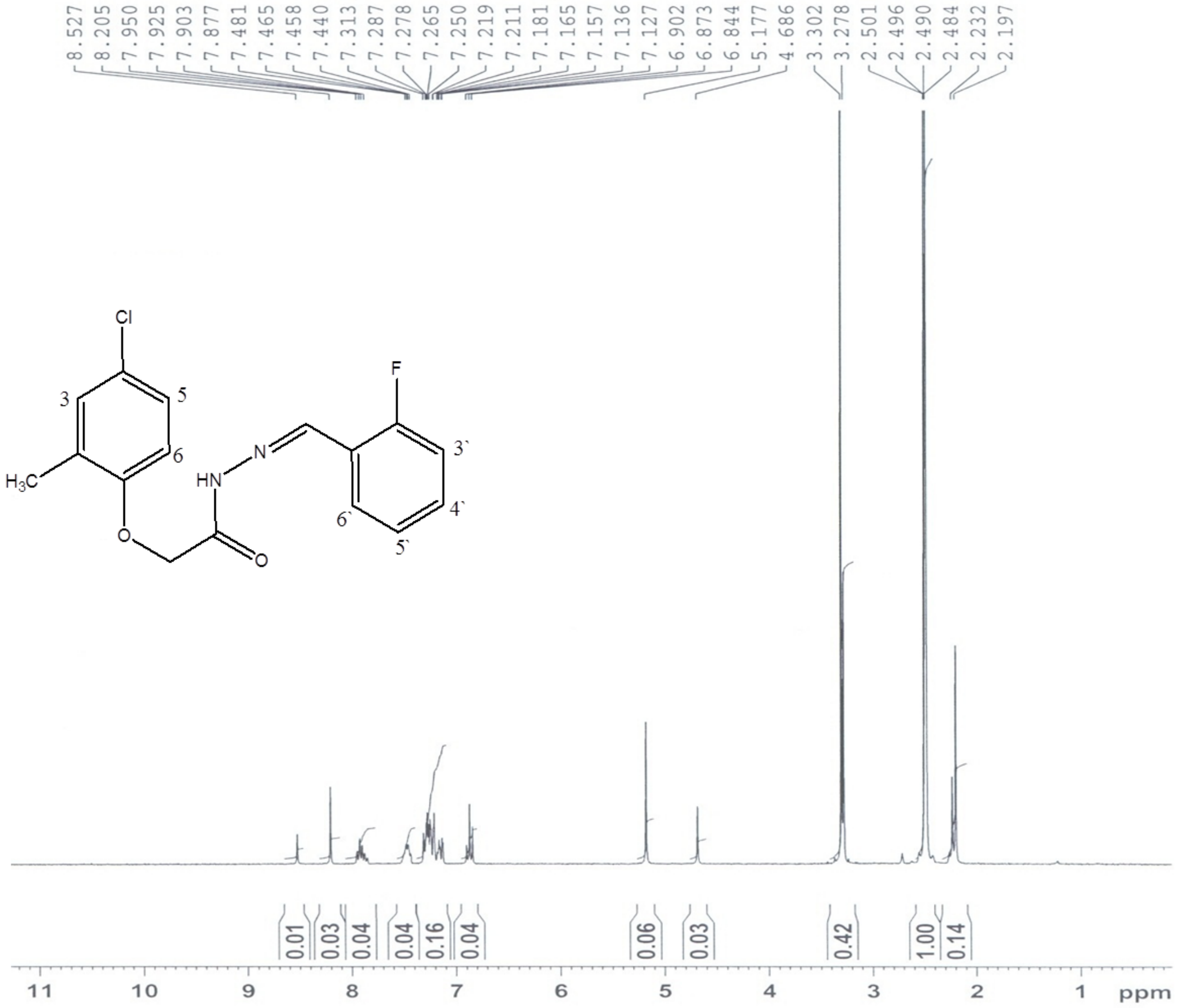

Figure S12. 1H-NMR spectrum (300 MHz, DMSO-d_6) of compound **12**.

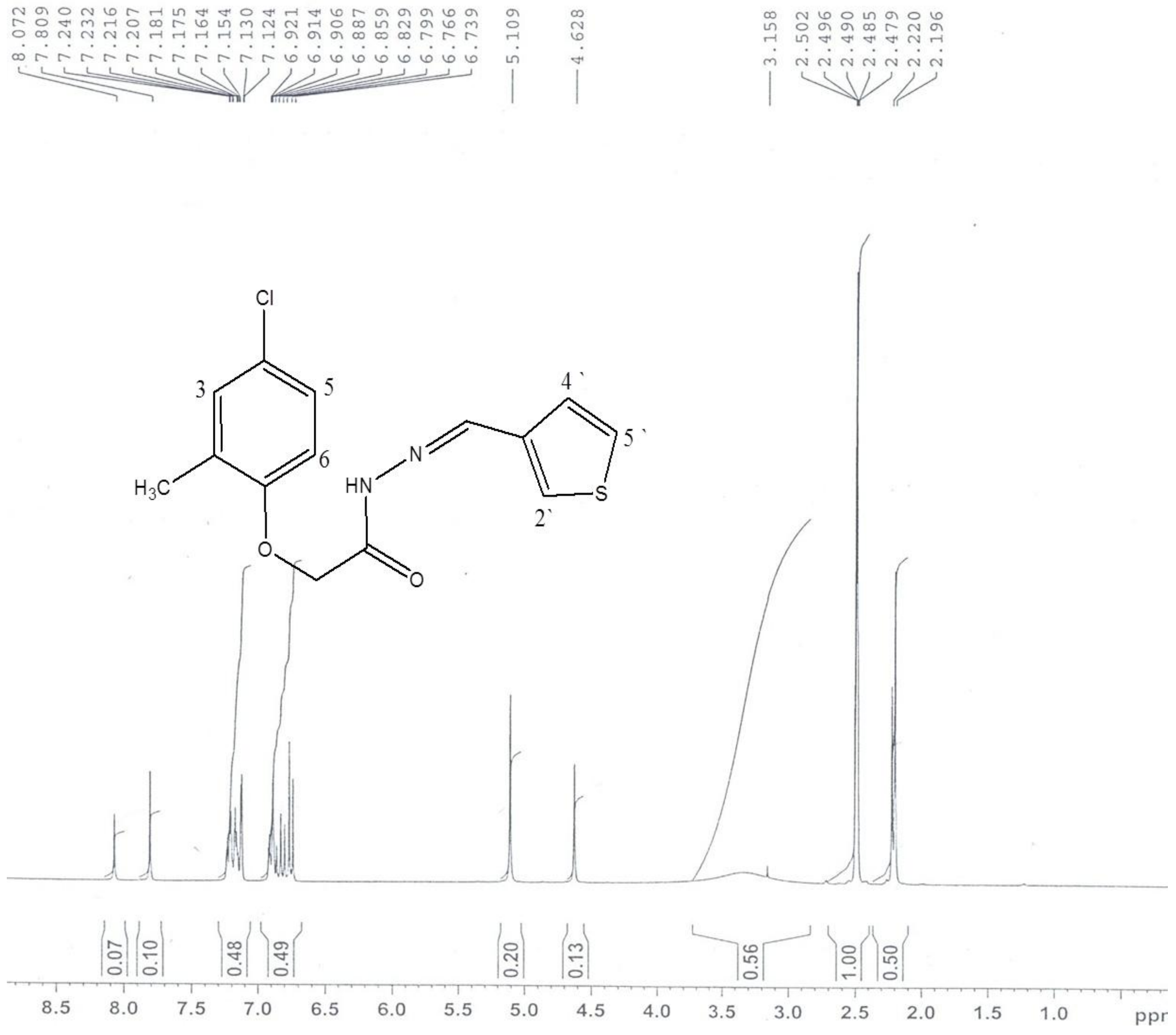

Figure S13. 1H-NMR spectrum (300 MHz, DMSO-d_6) of compound 1**3**.

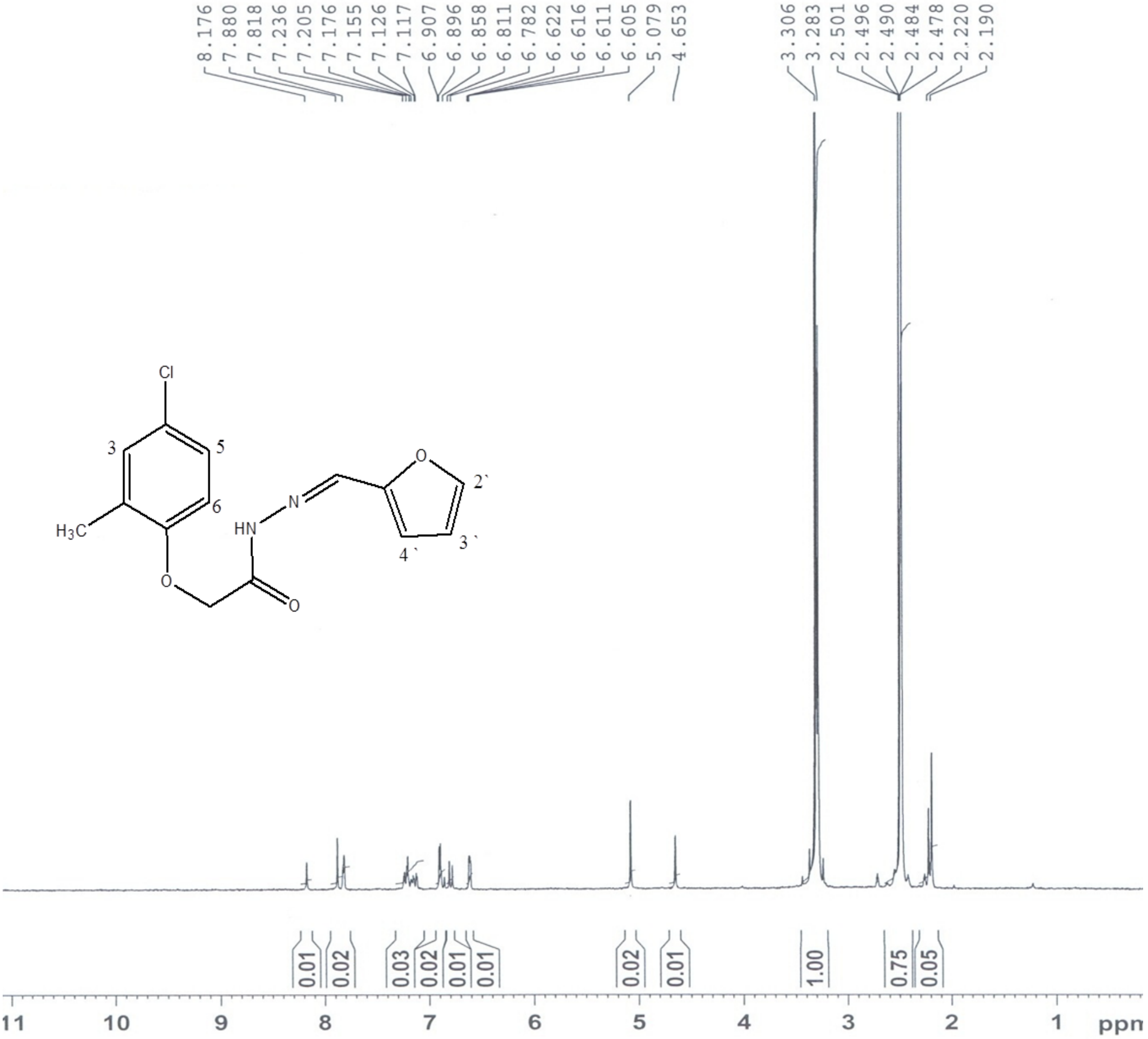

Figure S14. 1H-NMR spectrum (300 MHz, DMSO-d_6) of compound **14**.

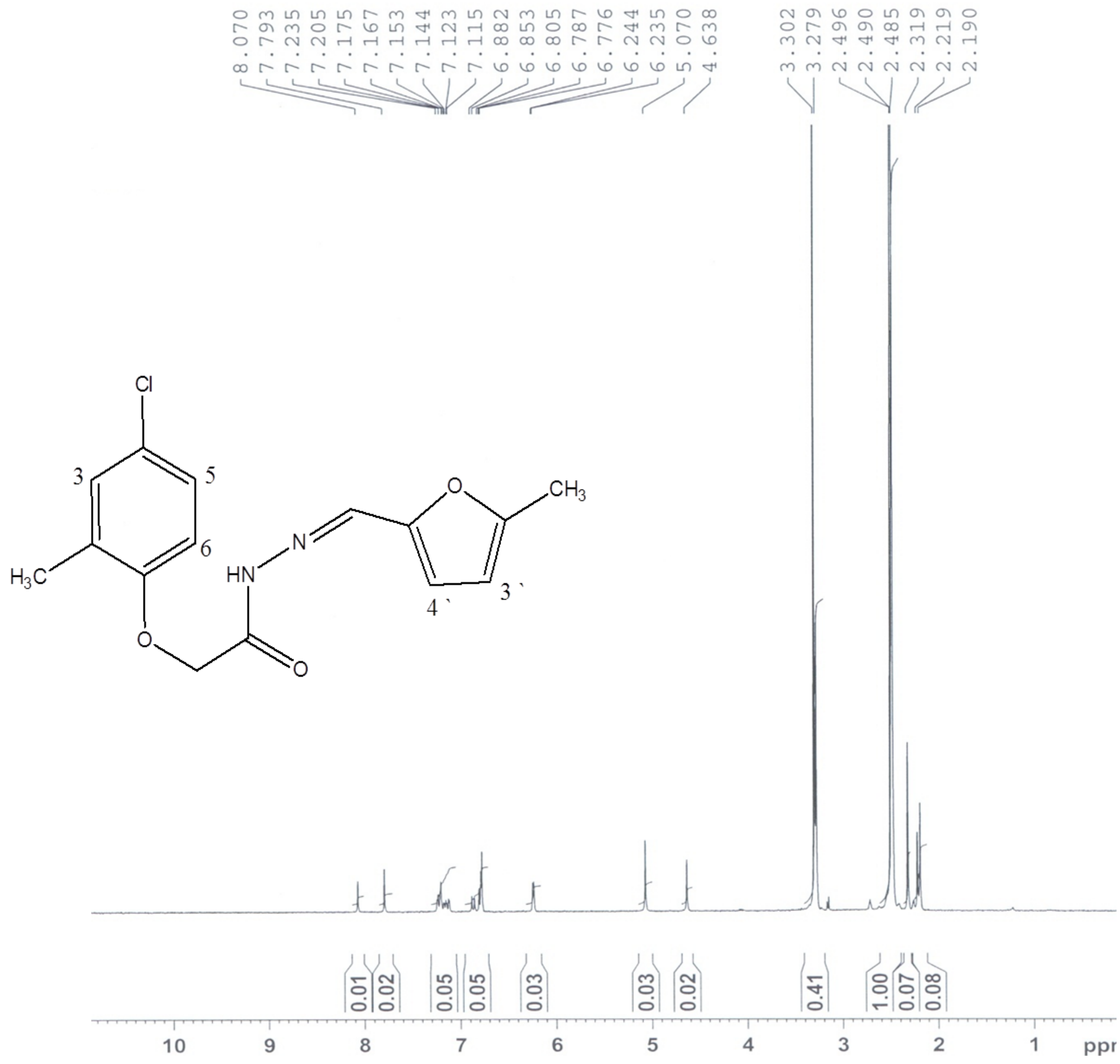

Figure S15. 1H-NMR spectrum (300 MHz, DMSO-d_6) of compound **15**.

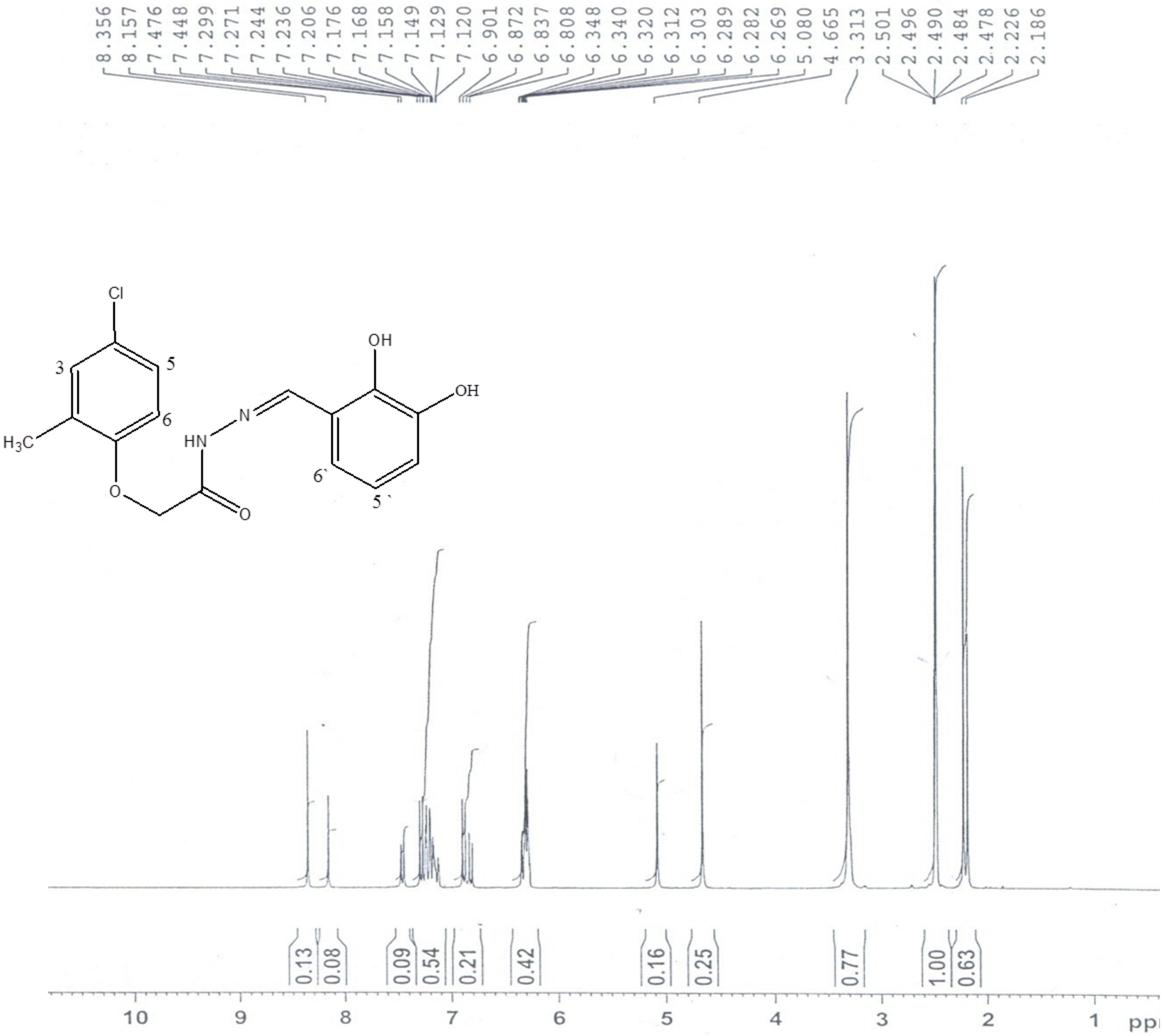

Figure S16. 1H-NMR spectrum (300 MHz, DMSO-d_6) of compound **16**.

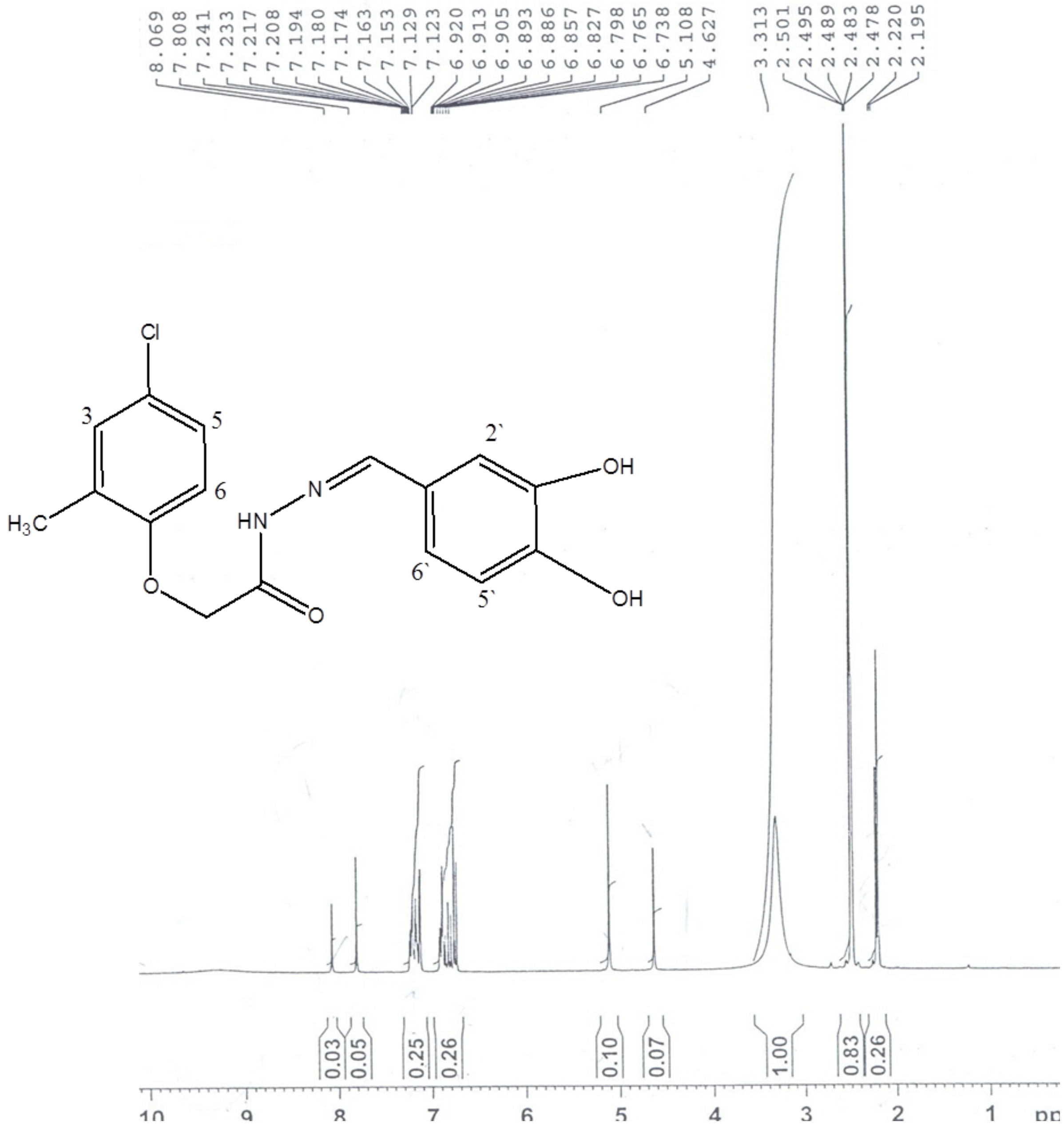

Figure S17. 1H-NMR spectrum (300 MHz, DMSO-d$_6$) of compound **17**.

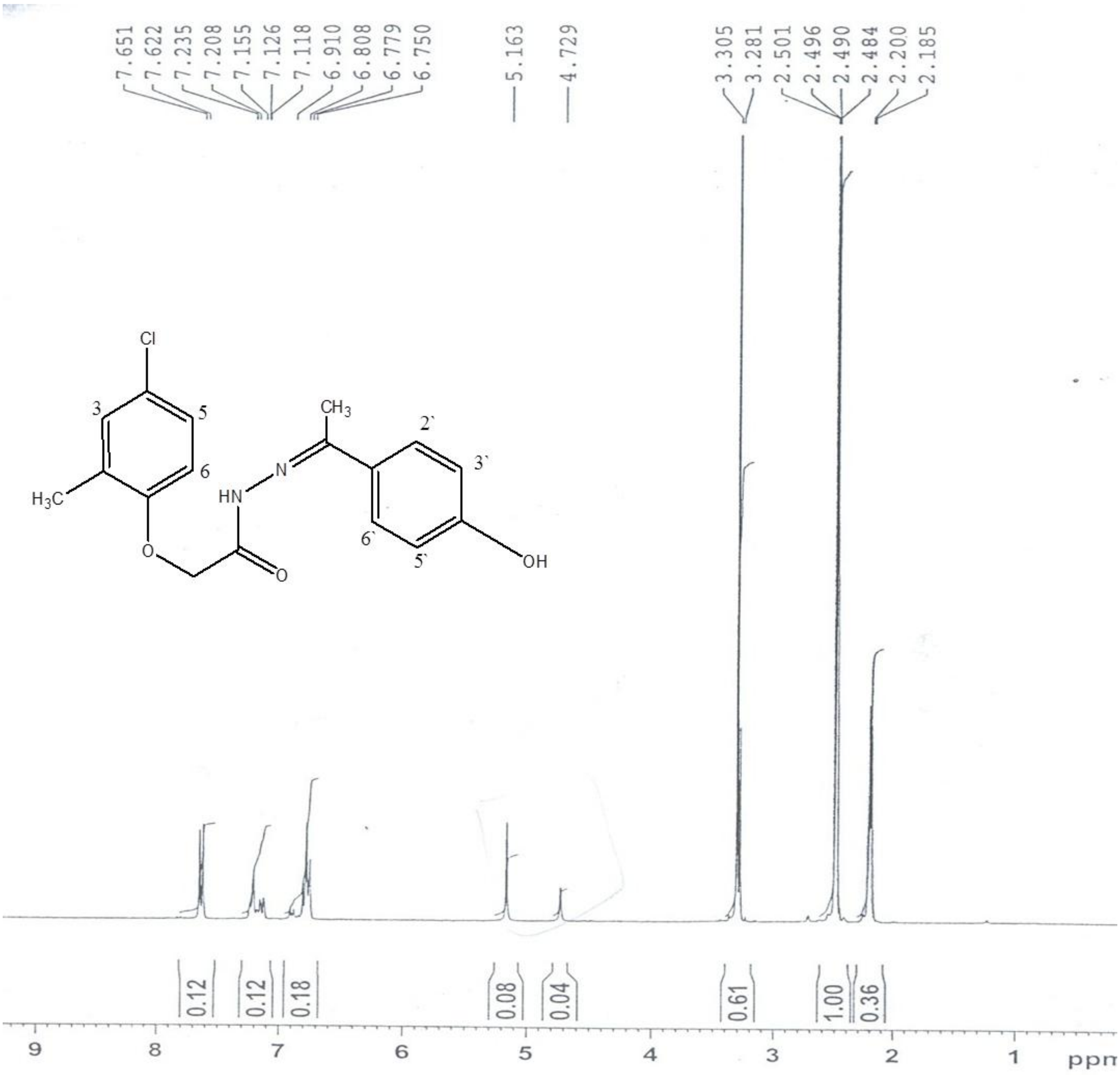

Figure S18. 1H-NMR spectrum (300 MHz, DMSO-d_6) of compound **18**.

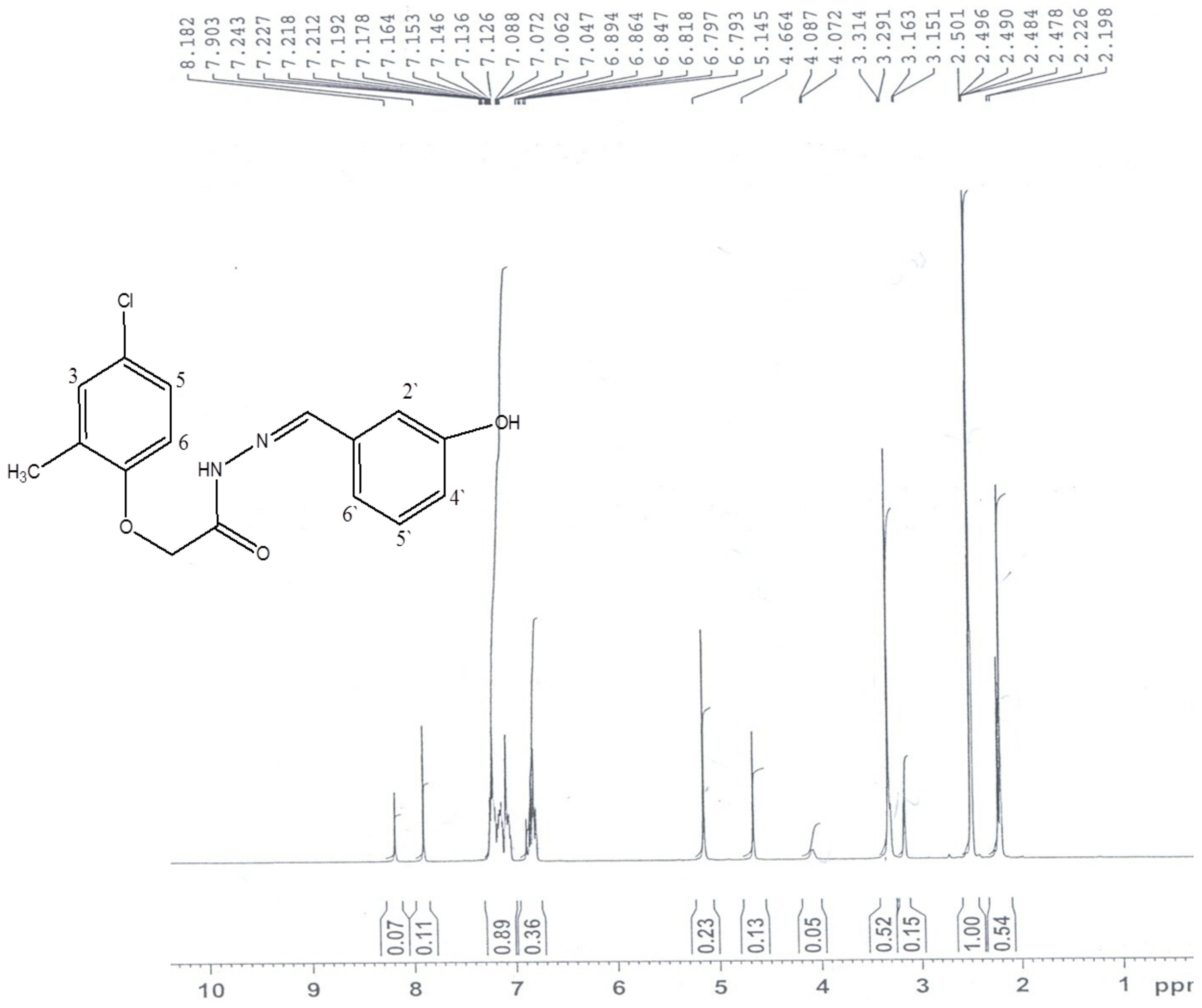

Figure S19. 1H-NMR spectrum (300 MHz, DMSO-d$_6$) of compound **19**.

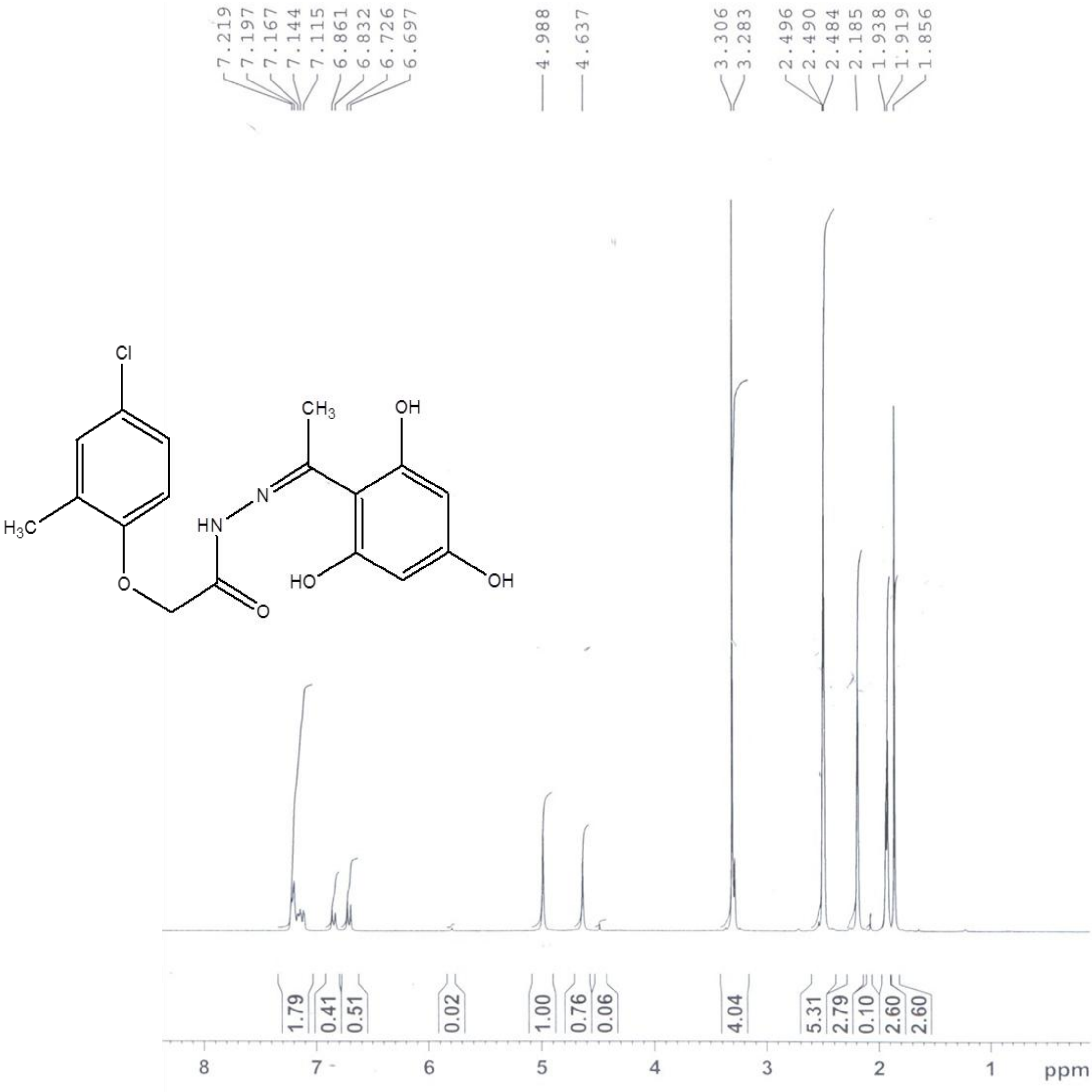

Figure S20. 1H-NMR spectrum (300 MHz, DMSO-d_6) of compound **20**.

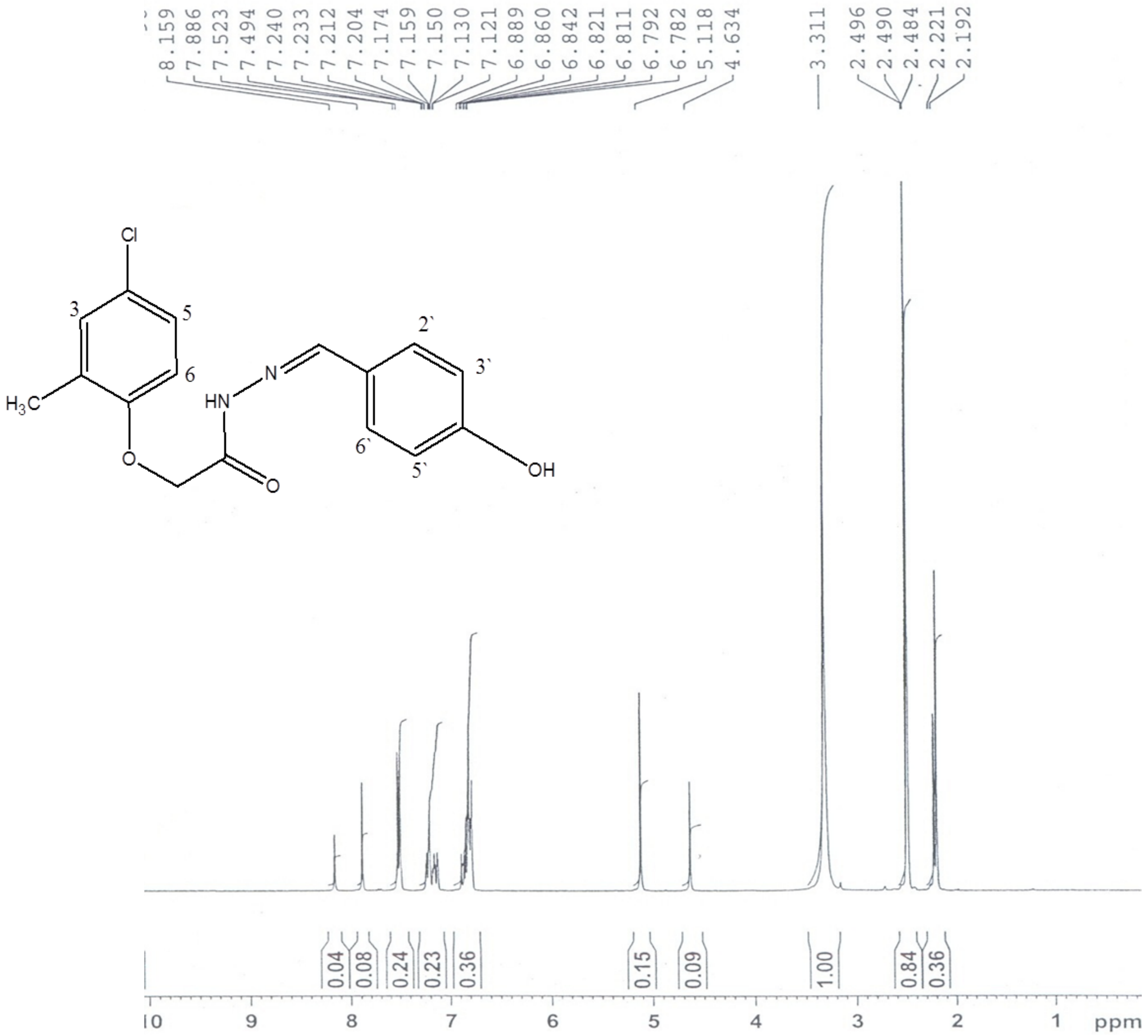

Figure S21. 1H-NMR spectrum (300 MHz, DMSO-d_6) of compound **21**.

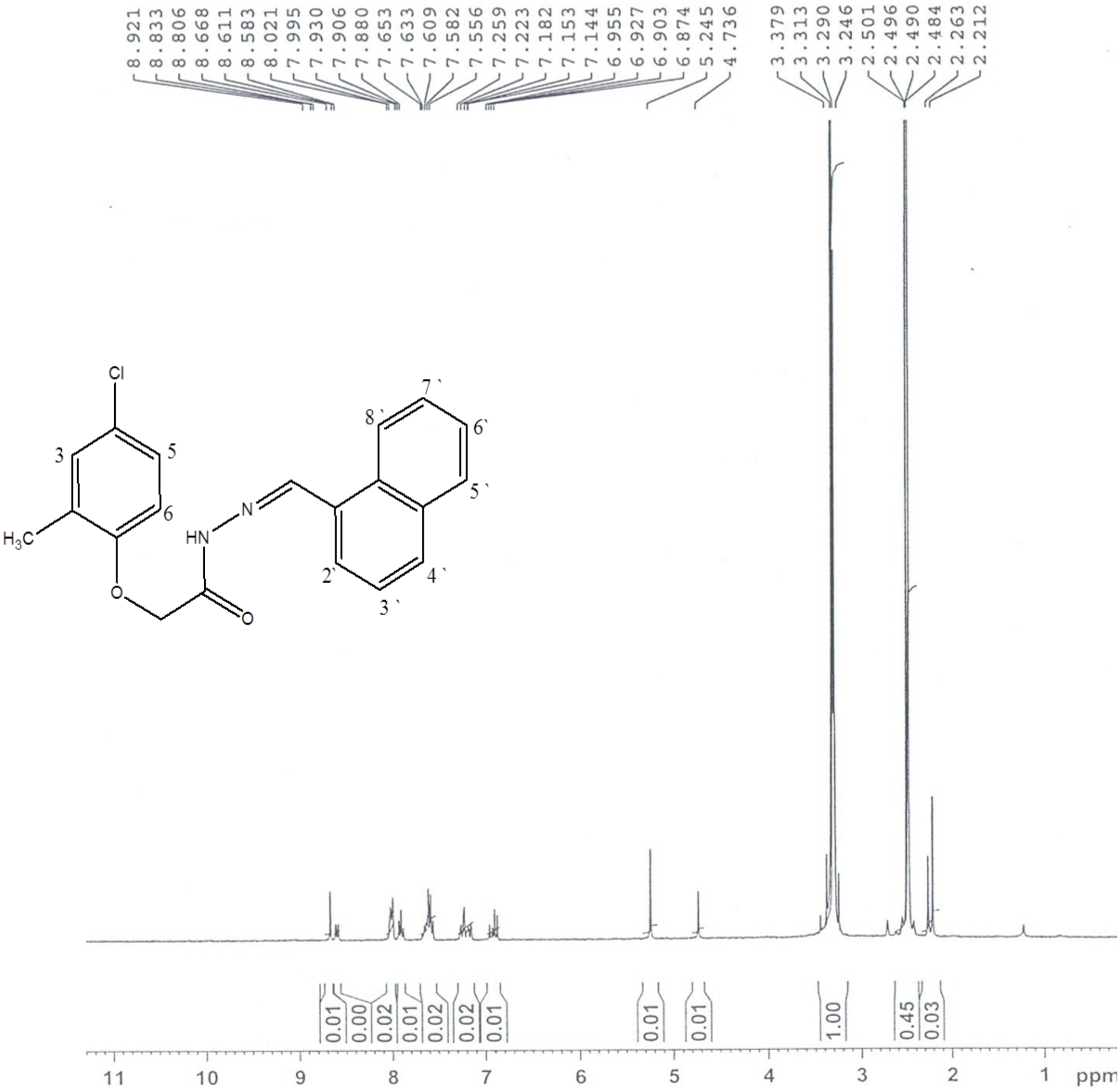

Figure S22. 1H-NMR spectrum (300 MHz, DMSO-d_6) of compound **22**.

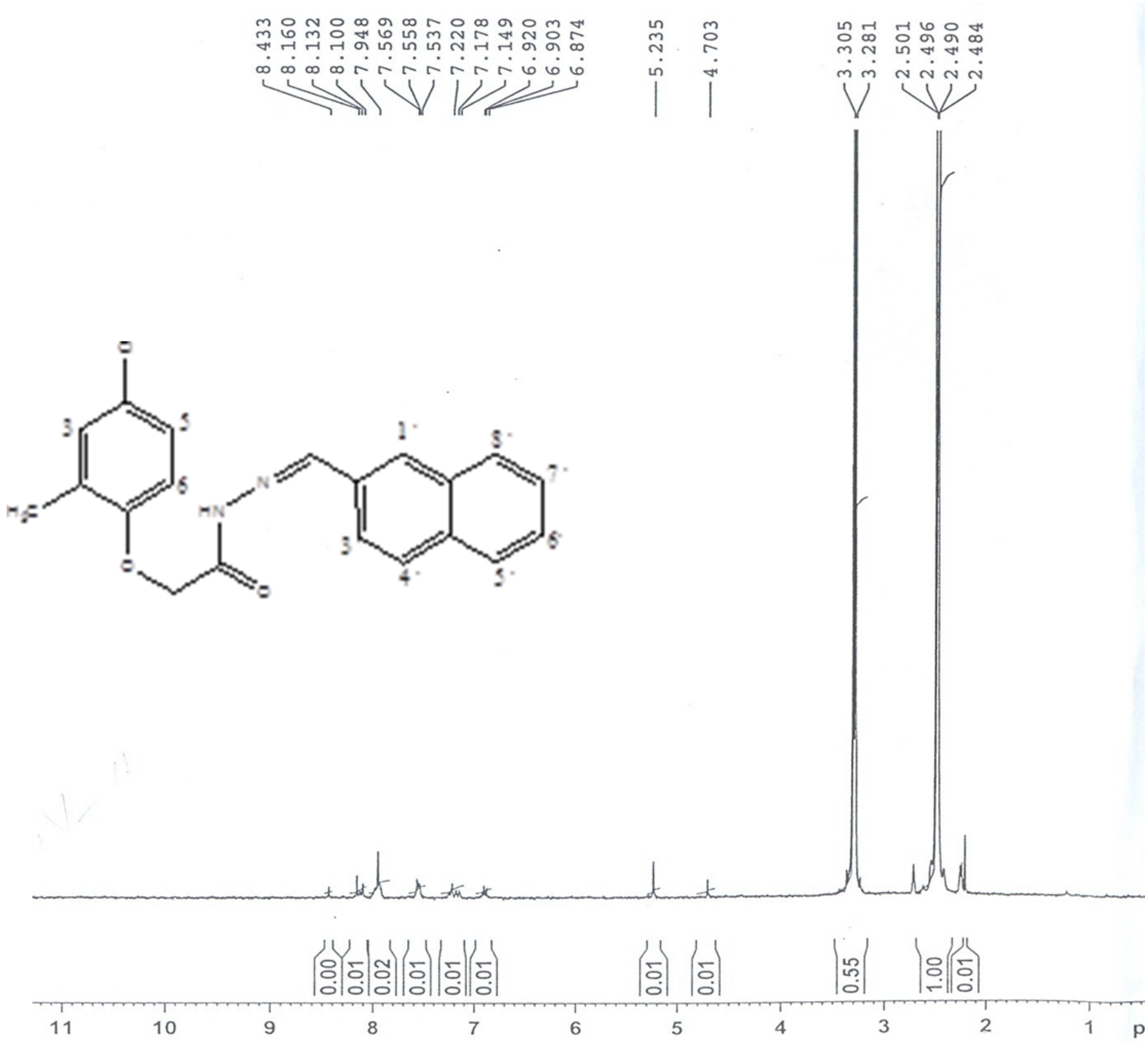

Figure S23. 1H-NMR spectrum (300 MHz, DMSO-d$_6$) of compound **23**.

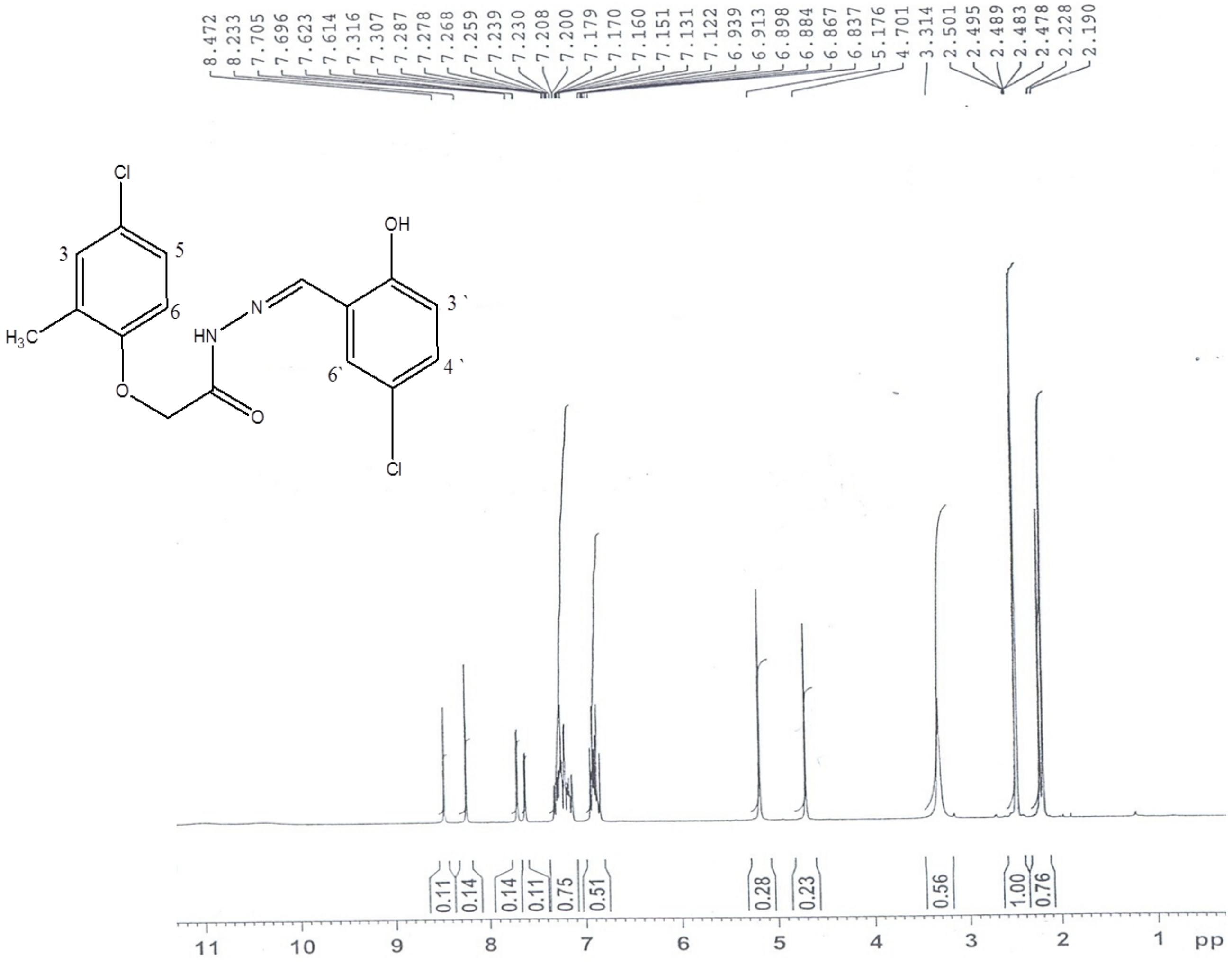

Figure S24. 1H-NMR spectrum (300 MHz, DMSO-d_6) of compound **24**.

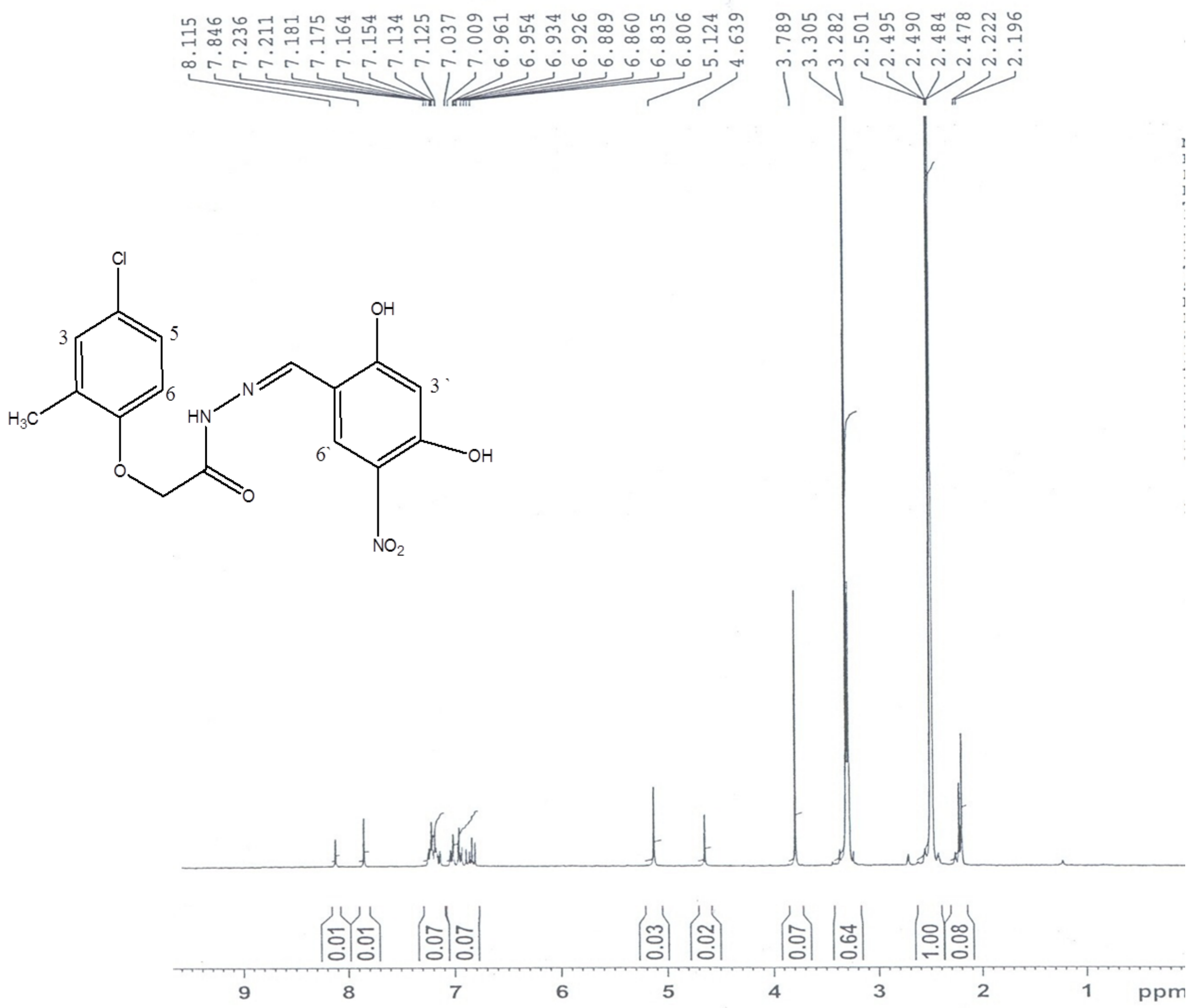

Figure S25. 1H-NMR spectrum (300 MHz, DMSO-d_6) of compound **25**.

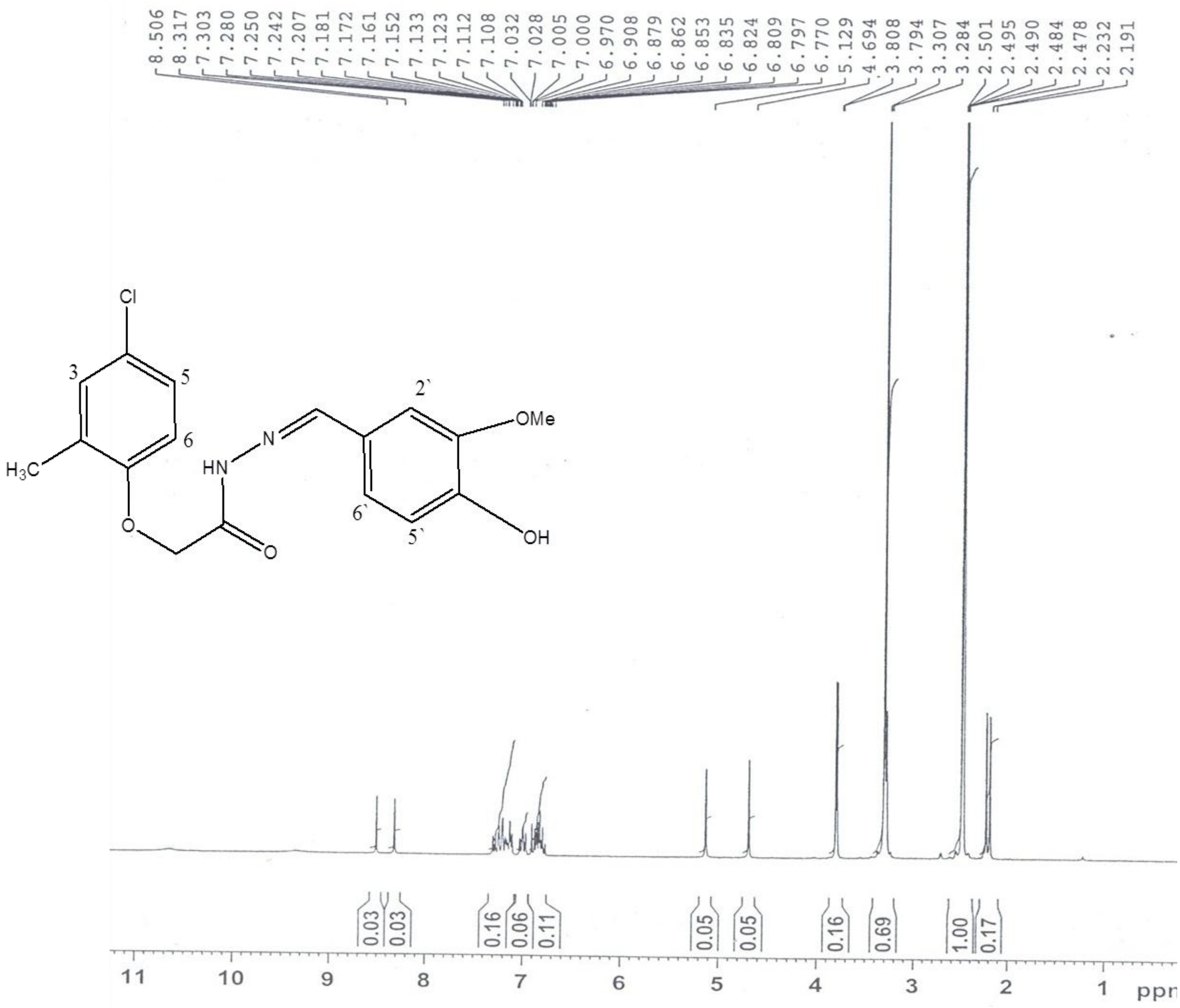

Figure S26. 1H-NMR spectrum (300 MHz, DMSO-d_6) of compound **26**.

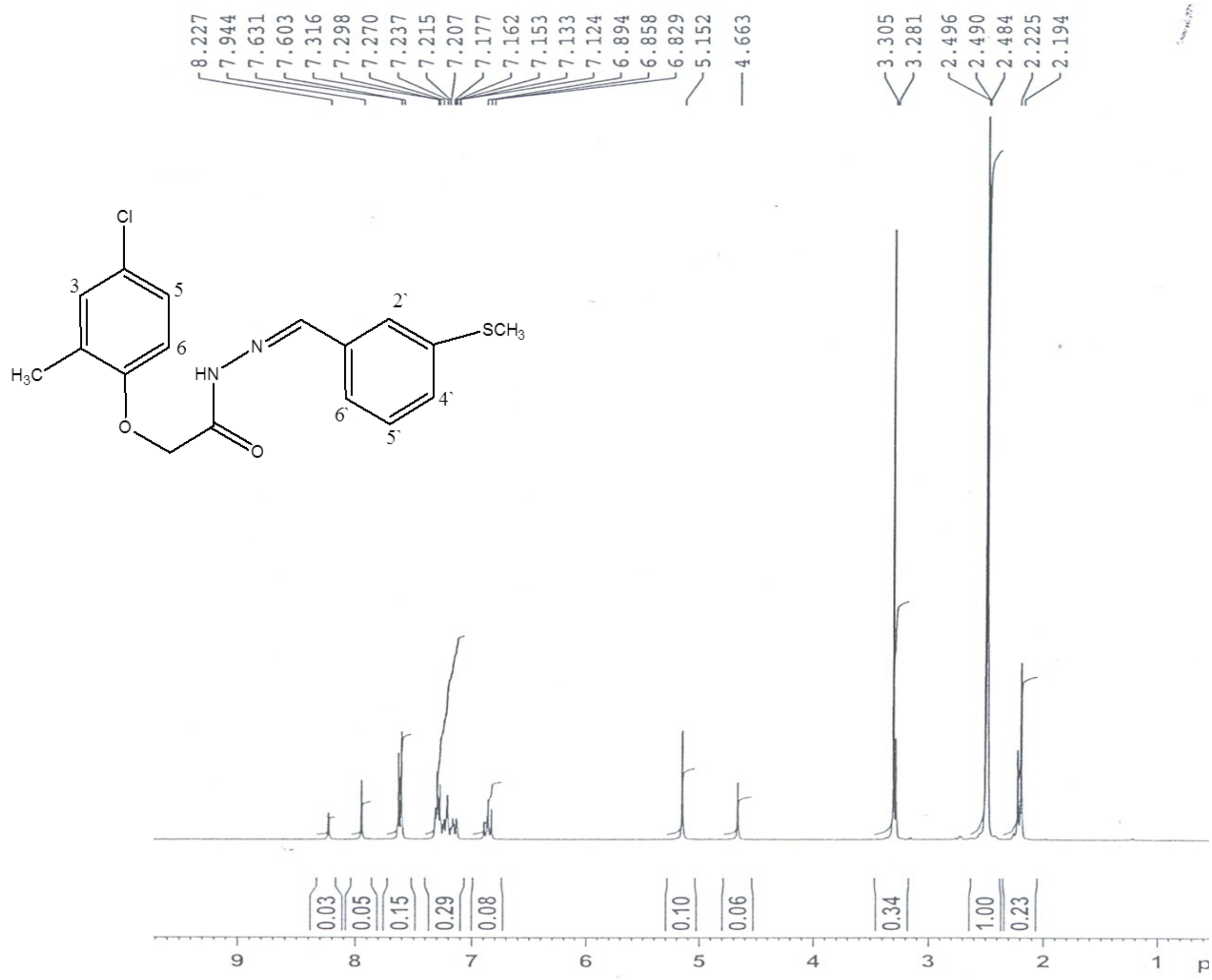

Figure S27. 1H-NMR spectrum (300 MHz, DMSO-d_6) of compound **27**.

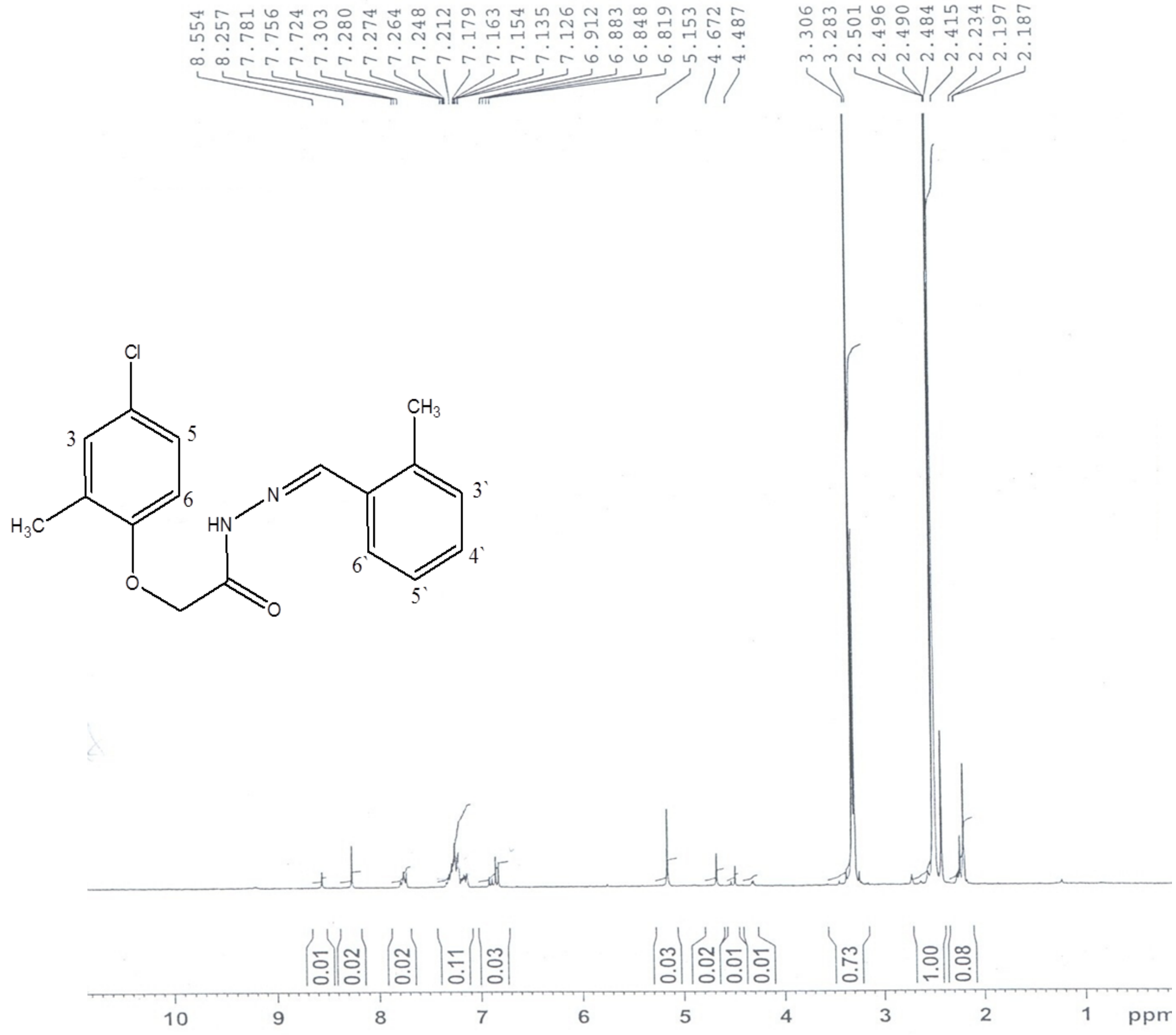

Figure S28. 1H-NMR spectrum (300 MHz, DMSO-d_6) of compound **28**.

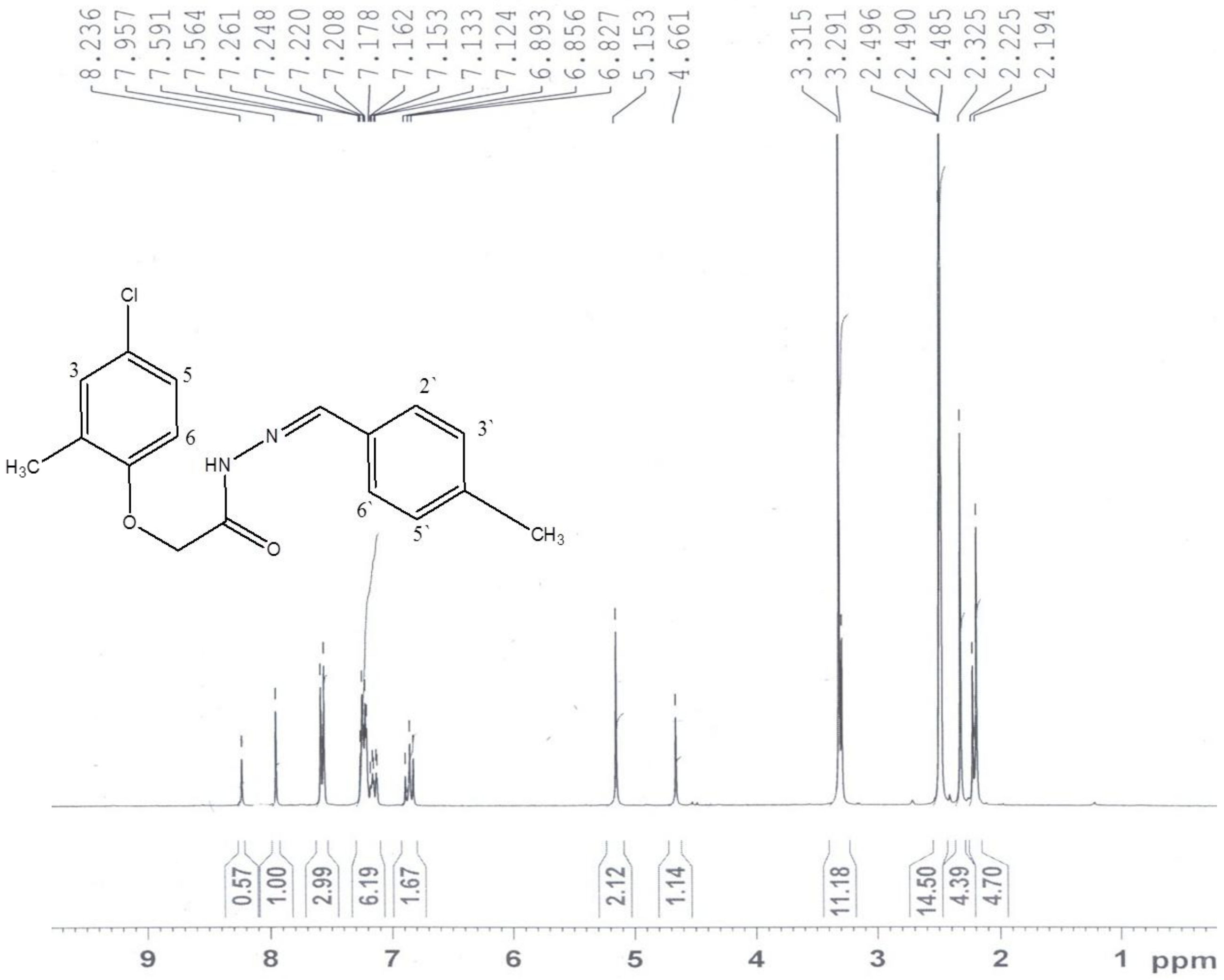

Supplement: Supplementary file 1 [file molecules-19-08788-s001.pdf]
